# Shared Control of a Powered Exoskeleton and Functional Electrical Stimulation Using Iterative Learning

**DOI:** 10.3389/frobt.2021.711388

**Published:** 2021-11-03

**Authors:** Vahidreza Molazadeh, Qiang Zhang, Xuefeng Bao, Brad E. Dicianno, Nitin Sharma

**Affiliations:** ^1^ Department of Mechanical Engineering and Material Science, University of Pittsburgh, Pittsburgh, PA, United States; ^2^ Neuromuscular Control and Robotics Lab, Joint Department of Biomedical Engineering, North Carolina State University and the University of North Carolina Chapel-Hill, Raleigh, NC, United States; ^3^ Department of Biomedical Engineering at University of Wisconsin-Milwaukee, Milwaukee, WI, United States; ^4^ Department of Physical Medicine and Rehabilitation, School of Medicine and Department of Bioengineering, University of Pittsburgh, Pittsburgh, PA, United States

**Keywords:** exoskeleton, wearable robot, shared control, iterative learning, functional electrical stimulation (FES)

## Abstract

A hybrid exoskeleton comprising a powered exoskeleton and functional electrical stimulation (FES) is a promising technology for restoration of standing and walking functions after a neurological injury. Its shared control remains challenging due to the need to optimally distribute joint torques among FES and the powered exoskeleton while compensating for the FES-induced muscle fatigue and ensuring performance despite highly nonlinear and uncertain skeletal muscle behavior. This study develops a bi-level hierarchical control design for shared control of a powered exoskeleton and FES to overcome these challenges. A higher-level neural network–based iterative learning controller (NNILC) is derived to generate torques needed to drive the hybrid system. Then, a low-level model predictive control (MPC)-based allocation strategy optimally distributes the torque contributions between FES and the exoskeleton’s knee motors based on the muscle fatigue and recovery characteristics of a participant’s quadriceps muscles. A Lyapunov-like stability analysis proves global asymptotic tracking of state-dependent desired joint trajectories. The experimental results on four non-disabled participants validate the effectiveness of the proposed NNILC-MPC framework. The root mean square error (RMSE) of the knee joint and the hip joint was reduced by 71.96 and 74.57%, respectively, in the fourth iteration compared to the RMSE in the 1st sit-to-stand iteration.

## 1 Introduction

Functional electrical stimulation (FES) is often prescribed to reanimate standing and walking functions in people with spinal cord injury (SCI) and other gait disorders due to stroke, multiple sclerosis, etc. (Chang et al. (2016); Nagai et al. (2016); Bulea et al. (2013)). FES is usually applied transcutaneously *via* adhesive electrode pads that deliver electrical currents to the skeletal muscles’ motor units. Electrical stimulation of the lower-limb muscles, when coordinated, can produce desired standing and walking movements. However, FES causes a rapid onset of muscle fatigue (Bickel et al. (2011)), which reduces the muscle’s ability to sustain or produce contraction force and significantly decreases the duration of FES-elicited tasks.

FES can be combined with a passive orthosis (Sharma et al. (2014); Bao et al. (2016); Alouane et al. (2019)) to alleviate the effects of FES-induced muscle fatigue. These hybrid devices lock knee joints during stance or standing to reduce FES stimulation duration but may not provide additional torque to the knee joints. Battery-operated powered exoskeletons (Strausser and Kazerooni (2011); Neuhaus et al. (2011); Farris et al. (2014)) can also supplement FES-elicited joint torque (del Ama et al., 2014); Ha et al. (2015); Kirsch et al. (2014); Alibeji et al. (2018b)). This combination, which is also known as a hybrid exoskeleton, can overcome FES limitations. Supplementing FES-induced muscle contractions with robotic assistance reduces the overall stimulation duty cycle, delaying the onset of muscle fatigue during high torque–demanding physical exercises like sit-to-stand tasks. The shared use may also reduce actuator size and power consumption in the powered exoskeleton. More importantly, unlike powered exoskeletons that passively move the limbs, FES-induced active muscle contractions contribute to neuroplasticity that may recover the lost limb function (Popovic et al. (2012)). The use of FES also promotes or improves bone health, overall limb elasticity, and cardiovascular and metabolic benefits (Peckham and Knutson (2005)). Thus, its integration with an exoskeleton system is likely to maintain or enhance the therapeutic benefits.

Despite its promising benefits, the hybrid exoskeleton’s dynamic shared control is an open research topic. Actuation redundancy due to FES and electrical motors’ simultaneous use and modulation of the shared effort to compensate for FES-induced fatigue dynamics are challenging control problems. Recent research efforts in this direction certainly inform ways to implement shared control in a hybrid exoskeleton, but these control designs did not explicitly account for FES-induced fatigue dynamics in functionally relevant and multi-DOF lower-limb movements. In the study by Quintero et al. (2012), the authors used an adaptive control method to allocate inputs to motors and FES. In studies by both (del Ama et al., 2014) and (Ha et al. (2015), a combination of feed-forward learning control and proportional-integral-derivative (PID) feedback controlled electric motors and FES. Optimal control is also a suitable approach for cooperative control of FES and an electric motor in the hybrid exoskeleton. Kirsch et al. (2018), Bao et al. (2016), and Bao et al. (2019) optimally controlled a one–degree-of-freedom (DOF) hybrid leg extension machine using a nonlinear model predictive control (NMPC) method to modulate FES and electric motor assistance as per the FES-induced fatigue dynamics. However, a muscle fatigue–based dynamic effort distribution between FES and an electric motor has not been attempted in functionally relevant and multi-DOF lower-limb movements. As a step toward this direction, this study aims to show the feasibility of a low-level optimal MPC strategy to dynamically distribute a higher-level knee torque between FES and the electric motor during sit-to-stand tasks.

For controlling different muscle groups and multiple electric motors during walking or swing-like leg movements, a muscle synergy–inspired controller used a set of synergy blocks in the works of Alibeji et al. (2017), Alibeji et al. (2015), and Alibeji et al. (2018a). Each synergy acted as a set of allocation ratios for different actuators. A modified PD controller provided robustness to modeling uncertainties, and a robust adaptive term modified the coefficients of a combination of synergies to compensate for the muscle fatigue. The muscle synergy–inspired controllers in the studies by Alibeji et al. (2017, 2015, 2018a) enabled automatic allocation of effort between the powered exoskeleton and FES and have been shown to provide good performance. However, the muscle synergy–inspired control design did not employ a real-time optimal control approach, and even then, it is not yet shown if an optimal control allocation can be embedded into a robust control framework that guarantees desired joint torque levels or system stability despite modeling uncertainties and disturbances.

Due to a lack of optimal control allocation strategies for the hybrid exoskeleton for sit-to-stand, or any functional task in general, this study explored the use of an MPC strategy to allocate FES and electric motor torques. The MPC strategy in this study hinges on a higher-level desired torque generator. However, unlike the techniques discussed in the study by Zhang et al. (2015) that generate torque based on predetermined angles, electromyography, or time, we use a novel neural network (NN)-based position tracking control approach to generate desired joint torques. The NN-based control approach and the associated NN update laws use a discrete Lyaupunov-like stability analysis that shows asymptotic error convergence for the first time for iterative sit-to-stand tasks. The NN-based control approach is robust to modeling uncertainties and time-varying disturbances in the FES-driven musculoskeletal dynamics. Notably, the NN-based control approach is derived to iteratively increase the feed-forward learning component and decrease the high-level torque generator’s high gain feedback component. The feed-forward learning is an improvement over our recent approach that used a high-gain position tracking controller for high-level torque generation for an experimental study on sit-to-stand tasks (Bao et al. (2020a)). Unlike most exoskeleton controllers that follow a time-dependent desired joint trajectory (Ha et al. (2012); Contreras-Vidal et al. (2016); Alibeji et al. (2018b); Bae and Tomizuka (2012)) or a desired time-dependent or EMG-generated torque trajectory (Zhang et al. (2015)), the designed NN-based approach follows state-dependent desired joint trajectories known as virtual constraints (Westervelt et al. (2007); Gregg and Sensinger (2014)).

Compared to our previous simulation studies by Molazadeh et al. (2019), Bao et al. (2020b), and Molazadeh et al. (2018a,b), the study presents a more detailed derivation of the controller, improved robustness to modeling uncertainties, and supporting stability analysis. Furthermore, extensive sit-to-stand experiments with a hybrid exoskeleton validated the approach on four participants with no disabilities. The experiments validated the proposed bi-level control framework for sharing control between the powered exoskeleton and FES dynamically.

The article is organized as follows. Section 2 describes the overall shared control design, which is followed by Section 3, which presents the experimental results, followed by discussion in Section 4 and the conclusion in Section 5. Supplementary Appendices A–D provide a more detailed open-loop and closed-loop error dynamics development, the stability analysis, virtual constraint design, and the MPC allocation algorithm, respectively.

## 2 Shared Control Framework

The main task of our control framework is to implement a learning control approach that estimates unknown/uncertain dynamics in an iterative fashion and then use the estimates in a controller that outputs stabilizing torques for a desired movement. Therefore, in the first subsection, an NNILC method is presented as a top-level controller. The NNILC method estimates the unknown/uncertain dynamics, and based on these estimates, it provides robust and stabilizing torques for a low-level controller. Furthermore, in the next subsection, a model predictive approach is used as the low-level controller to distribute the NNILC-designed torque among FES and the powered exoskeleton. This control framework can be used for repetitive movements including repeated sit-to-stand and walking. Below, we first present a general hybrid exoskeleton model.

An N-DOF hybrid exoskeleton that comprises N_f_ muscles where FES is applied, N_m_ electric motors, and a powered exoskeleton is modeled as follows:

$$M\left(\theta \right)\ddot{\theta }+C\dot{\theta }+G+T_{p}=T+D,$$ (1)

where $\theta \in R^{N}$ , $\dot{\theta }\in R^{N}$ , and $\ddot{\theta }\in R^{N}$ are the vectors that represent the links’ angular position, angular velocity, and angular acceleration, respectively, $M\left(\theta \right)\in R^{N\times N}$ is the inertia matrix, $C\left(\dot{\theta },\theta \right)\in R^{N\times N}$ is the centripetal-Coriolis matrix, $G\left(\theta \right)\in R^{N}$ is the gravitational vector, $D\in R^{N}$ is the system disturbance, $T_{p}\in R^{N}$ is the passive viscoelastic moment, and $T\in R^{N}$ is the combined torque generated due to FES and the powered exoskeleton.

### 2.1 Top-Level Control Structure

In this subsection, a top-level controller is presented. Its detailed derivation using the open-loop and closed-loop error dynamics are provided in Supplementary Appendix A.

The control objective is to ensure that the independent joint angle function, $\theta \in R^{N}$ , in Eq. 1 follows a specially designed desired constraint function, $h\left(\theta \right)\in R^{N}$ , which is a function of the system state. Usually, h is solely a function of time, for example, a time-dependent desired trajectory, θ_d_(t). Instead, we chose to design the desired trajectory as a state-dependent trajectory. This state-dependent design is motivated to constrain the desired movement of multiple joints to a single joint (phase variable). The phase variable must be state-dependent and a monotonically increasing function. The advantage of this approach is that it avoids joints miscoordination that may be caused by using multiple time-dependent joint-desired trajectories. The method to design the desired constraint function is given in Supplementary Appendix C. The control objective can be expressed as an output, $y\in R^{N}$ ,

$$y_{k}≜\theta_{k}-h_{k},$$ (2)

which must be driven to zero. Subscription k shows the value of the variables in the k^th^ iteration (e.g., in a repetitive sit-to-stand task, each sit-to-stand can be considered as one iteration).

The sliding surface $s_{k}\in R^{N}$ is designed as

$$s_{k}=\lambda_{1}e_{1,k}+\lambda_{2}e_{2,k},$$ (3)

where λ_1_ and λ_2_ are positive constants and e_1,k_ = − y_k_, $e_{2,k}=-{\dot{y}}_{k}$ .

Based on the stability analysis provided in Supplementary Appendix B, the top-level controller, *U* *_k_*, is designed as given below:

$$U_{k}=-{\hat{f}}_{2,k}-{\hat{\sigma }}_{k}f_{1,k}-F_{k},$$ (4)

where ${\hat{\sigma }}_{k}$ is an estimate of the parameter, σ in the output dynamics in (24). Based on the subsequent stability analysis in Supplementary Appendix B, ${\hat{\sigma }}_{k}$ is updated after each iteration as follows:

$${\hat{\sigma }}_{k}={\hat{\sigma }}_{k-1}-q_{c}f_{1,k}\gamma s_{k},$$ (5)

where ${\hat{\sigma }}_{k}=0\;when,k=-1$ , γ is a positive constant, and q_c_ is a positive constant that tunes the speed of updating ${\hat{\sigma }}_{k}$ . $F_{k}\in R^{N}$ is an additional feedback input, f_1,k_ is a known part of the dynamics (for details, see Supplementary Appendix A), and ${\hat{f}}_{2,k}$ is the approximation of the ideal NNs for the unknown/uncertain part of the output dynamics, f_2,k_ (for details, see Supplementary Appendix A). This approximation is represented as follows:

$${\hat{f}}_{2,k}={\hat{W}}_{k}^{T}\Lambda_{k}\left({\hat{P}}_{k}^{T}X_{k}\right),$$ (6)

where ${\hat{W}}_{k}\in R^{N_{2}+1\times N}$ and ${\hat{P}}_{k}\in R^{\left(2N+1\right)\times N_{in}}$ are the estimates of ideal weights in (26) in the k^th^ iteration. These estimates are updates using gradient-based laws. Their update laws are provided in Supplementary Appendix A ((28) and (29)).

F_k_ in Eq. 4 is designed as follows:

$$\begin{array}{c} F_{k}=\frac{1}{\lambda_{2}}\left(\lambda_{1}{\dot{y}}_{k}-\lambda_{2}\left(\alpha_{2}s_{k}+\frac{4}{3}\alpha_{1}\mathrm{sgn}\left(s_{k}\right)\right)+\lambda_{2}I_{k}\right), \end{array}.$$ (7)

where $\alpha_{1}\in R^{+}$ and $\alpha_{2}\in R^{+}$ are control gains, $\lambda_{1},\;\lambda_{2}\in R^{+}$ are constant values, and *I* *_k_* is a low pass filter term that is designed as follows:

$${\dot{I}}_{k}=-\beta_{1}s_{k}-\beta_{2}I_{k},$$ (8)

where β_1_ and β_2_ are positive constants. The term F_k_ has been added to the top-level control input to keep the closed-loop system stable when considerable estimation errors may be present during initial iterations.

Remark 1. Because the top-level controller is based on NNs and updates itself every iteration, we call it the NN-based ILC. Choosing its control parameters has significant effect on the learning speed in each iteration and the closed-loop control system performance. For example, the speed of learning of σ can be changed by q_c_ in Eq. 5. Similarly the speed of learning of f_2_ can be changed by changing the learning gains in the weight update laws. These gains are subsequently defined as ρ_1_ and ρ_2_ in (29) and (28) in Supplementary Appendix A. Please note that the speed of learning must not be chosen to be so high that it causes destabilization of the closed-loop control system and/or noise accumulation.

### 2.2 Predictive Allocation Strategy

In this subsection, a lower-level controller is formulated that determines the allocation of control between motors and FES. Mainly, the objective of the low-level controller is to constrain the optimized FES and the electric motor torque values to the desired torque level that is dictated by the top-level controller in the first subsection. An MPC allocation strategy is used for this purpose. A strategy is also designed to consider the muscle fatigue level by including a fatigue variable as a weighting variable in the cost function. The optimization objective is to minimize a cost function. $J_{mpc}\left(t_{r}\right)\in R^{+}$

$$\begin{array}{ccc} & \underset{\;\;\;\;\;\;{\bar{u}}_{M,k},\;{\bar{u}}_{F,k}}{\;\;\;\;\;\;\text{min}}\;J_{mpc}\left(t_{r}\right)=\int_{t_{r}}^{t_{r}+t_{p}}\left\{{\bar{T}}_{M,k}^{T}w_{1}{\bar{T}}_{M,k}+{\bar{T}}_{F,k}^{T}w_{2}{\bar{T}}_{F,k}\right\}dt & \end{array}$$ (9)

$$\begin{array}{ccc} & \text{s.t.}\;\;M\left({\bar{\theta }}_{k}\right){\ddot{\bar{\theta }}}_{k}+C\left({\bar{\theta }}_{k},{\dot{\bar{\theta }}}_{k}\right){\dot{\bar{\theta }}}_{k}+G\left({\bar{\theta }}_{k}\right)+{\bar{T}}_{p,k} & \\ & ={\bar{T}}_{M,k}+{\bar{T}}_{F,k} & \end{array}$$ (10)

$$\begin{array}{ccc} & B_{M}u_{M,k}\left(t_{r}\right)+\psi_{k}u_{F,k}\left(t_{r}\right)=U_{k}\left(t_{r}\right) & \end{array}$$ (11)

$${\bar{u}}_{F,j,k}\in U$$ (12)

where the terms with a bar, for example, $\bar{x}$ , represents the nominal variable that is evaluated in the prediction horizon. In Eq. 9, *u*_*M,k*_ represents the motor input and *u*_*F,k*_ is the FES input. In Eq. 11, $U\in \left[0,\;1\right]\times \left[t_{r},t_{r}+t_{p}\right]$ is the input constraint (Kirsch et al. (2018); Sun et al. (2018)). Subscription r is the receding horizon value, for example, t_r_ shows the time in the *r*th receding horizon. Subscription k shows the value of a variable in the k^th^ ILC update, which is considered as one sit-to-stand movement. During each ILC update, the optimization problem in Eq. 9 is solved to determine ${\bar{u}}_{\bar{M},k}$ and ${\bar{u}}_{\bar{F},k}$ . In Eq. 9, the motor torque ${\bar{T}}_{M,k}$ in the prediction horizon is evaluated as follows:

$${\bar{T}}_{M,k}=B_{\bar{M}}{\bar{u}}_{M,k},$$ (13)

where $B_{\bar{M}}$ is a vector of known motor constants. ${\bar{T}}_{F,k}$ in Eq. 9 is the torque input of FES in the prediction horizon, and the *j*th element of this vector is defined as follows (Kirsch et al. (2016)):

$${\bar{T}}_{F,j,k}=\varphi_{j}\left({\bar{\theta }}_{1,j,k},{\dot{\bar{\theta }}}_{1,j,k}\right){\bar{\mu }}_{j,k}{\bar{u}}_{F,j,k},$$ (14)

where ${\bar{u}}_{F,j,k}$ is the k^th^ iteration of the nominal value of *u*_*F,j,k*_ in Eq. 16, and $\varphi_{j}\left({\bar{\theta }}_{1,j,k},{\dot{\bar{\theta }}}_{1,j,k}\right)$ represents torque-angle and angular velocity relationships (Popović et al. (1999); Kirsch et al. (2016); Bao et al. (2020b)) that map the limb angle and angular velocities to the joint torque are defined as follows:

$$\varphi_{j}\left({\bar{\theta }}_{1,j,k},{\dot{\bar{\theta }}}_{1,j,k}\right)=\left(c_{2,j}{\bar{\theta }}_{1,j,k}^{2}+c_{1,j}{\bar{\theta }}_{1,j,k}+c_{0}\right)\left(1-c_{3,j}{\dot{\bar{\theta }}}_{1,j,k}\right).$$

${\bar{\theta }}_{1,j,k}$ is the nominal value of the *j*th joint angle, and $c_{0,j}\in R^{+},\;c_{1,j}\in R^{+},\;c_{2,j}\in R^{+}$ and $c_{3,j}\in R^{+}$ are muscle parameters. ${\bar{\mu }}_{j,k}$ is evaluated using a differential equation that is used for the estimation of the current fatigue level in the studies by Riener et al. (1996) and Kirsch et al. (2016). The differential equation is represented as follows:

$${\dot{\bar{\mu }}}_{j,k}=\frac{\left(\mu_{min,j}-{\bar{\mu }}_{j,k}\right){\bar{u}}_{F,j,k}}{\tau_{f,j}}+\frac{\left(1-{\bar{\mu }}_{j,k}\right)\left(1-{\bar{u}}_{F,j,k}\right)}{\tau_{r,j}},$$ (15)

where $\mu_{min,j}\in \left[0,1\right)$ is the minimum fatigue level of the targeted muscle, $\tau_{f,j}\in R^{+}$ is the fatigue time constant, and $\tau_{r,j}\in R^{+}$ is the recovery time constant. ψ_k_ and the constraint (11) are defined and further developed in the next subsection.

$w_{1}\in R^{N\times N}$ and $w_{2}\in R^{N\times N}$ in Eq. 9 are the diagonal weight matrices. w_1_ is a predefined constant matrix, but w_2_ is a nonconstant matrix and is dependent on the fatigue variable, $\bar{\mu }$ , that is, its *j*th diagonal element is $\frac{1}{{\bar{\mu }}_{j,k}+\epsilon_{j}}$ , where $\epsilon_{j}\in R^{+}$ is a constant.

The objective index $J_{mpc}\left(t_{r}\right)\in R^{+}\cup \left\{0\right\}$ in Eq. 9 depends on control allocation between ${\bar{T}}_{M,k}$ and ${\bar{T}}_{F,k}$ along the time horizon $\left[t_{r},\;t_{r}+t_{p}\right]$ , where t_p_ is the time horizon length and t_r_ is the current time. When the optimal solution, $u_{F,k}^{*}\left(\left.t\right|:\;t\in \left[t_{r},\;t_{r}+t_{p}\right]\right)=\mathrm{arg min}\left\{J_{mpc}\left(t_{r}\right)\right\}$ , is found, $u_{F,k}=u_{F,k}^{*}\left(\left.t\right|:\;t=t_{r}\to t_{r}+\varepsilon \right)$ is applied to the system, where ɛ is an infinitesimal time constant that makes *t_k+1_* *=* *t* *_r_* + ɛ (Graichen and Kugi (2010)). For details about the implementation of this algorithm, please refer to Supplementary Appendix D.

### Control Distribution Between Functional Electrical Stimulation and Motor

The distribution of the control effort between FES and the powered motor is described in this subsection. Based on the calculated optimal normalized FES virtual input, *u*_*F,k*_, through the MPC algorithm and (11), the motor input can be calculated using the following:

$$u_{M,k}=B_{M}^{-1}\left(U_{k}-\psi_{k}u_{F,k}\right)$$ (16)

where *U*_*k*_ is defined in Eq. 4, B_m_ is a known vector of motor constant gains, and *ψ*_*k*_ is given by the following:

$$\psi_{k}={\hat{B}}_{F,k}+\varrho$$ (17)

where the spectral radius of ${\hat{B}}_{F,k}$ , $\varrho \in R^{N^{+}}$ , is added to ψ_k_ in order to avoid a singularity when ${\hat{B}}_{F,k}$ is equal to zero (Chen et al. (2010)). ${\hat{B}}_{F,k}$ is the approximation of ideal NNs for uncertain control gain associated with normalized FES input, *B*_*F,k*_, and is expressed as follows:

$${\hat{B}}_{F,k}={\hat{Q}}_{k}^{T}\phi_{k}\left(X_{k}\right)$$ (18)

where ${\hat{Q}}_{k}\in R^{N_{\Omega }\times N}$ is the estimates of ideal weights in (27) in the k^th^ iteration. Based on the subsequent stability analysis provided in Supplementary Appendix B, it is updated according to the following update law

$${\dot{\hat{Q}}}_{j,k}=-\chi \phi_{j,k}\left(X_{k}\right)u_{F,j,k}s_{j,k}.$$ (19)

where $\chi \in R^{+}$ is a constant.

### 2.3 Overall Bi-Level Control Structure

**FIGURE 1:**
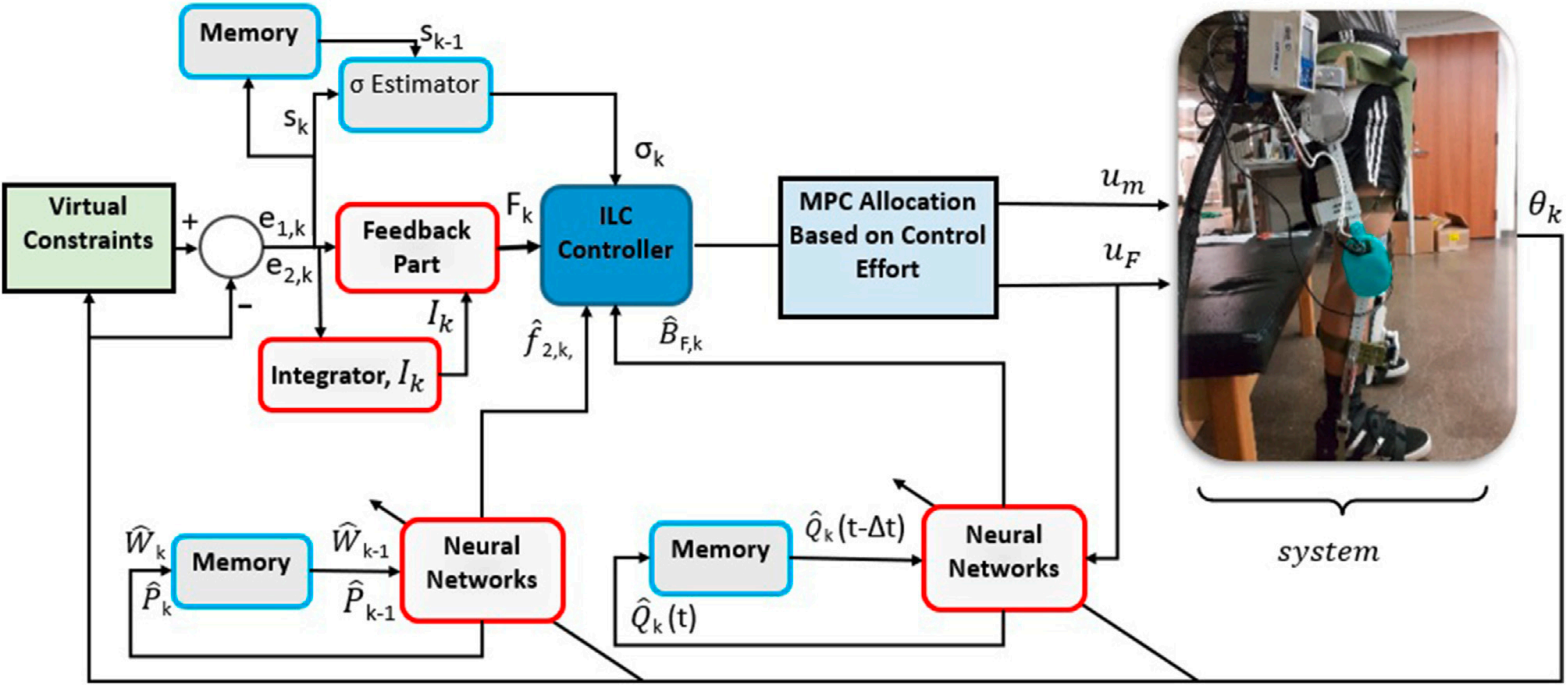
Structure of the proposed controller and the test bed that are used during experiments for the controller validation.

The control schematic is depicted in Figure 1.

As can be seen in this figure, the top-level ILC controller block uses three inputs: the NN estimates ${\hat{f}}_{2,k}$ and ${\hat{B}}_{F,k}$ , the linearly parameterizable adaptive component ${\hat{\sigma }}_{k}$ , and the feedback component *F*_*k*_. The total torque demand at the knee joint is allocated optimally using the low-level MPC method. Figure 1 shows how information flows between the blocks, information needed to train the NNs, and the interaction between the feedback and feed-forward parts of the control design. According to the figure, the linearly parameterizable part of the output dynamics, ${\hat{\sigma }}_{k}$ , is trained based on its value in the previous iteration, ${\hat{\sigma }}_{k-1}$ , and the value of the sliding surface, *s*_*k*_. Mathematical details can be found in Eq. 5. The figure also shows the training process of the FES control gain, ${\hat{B}}_{F,k}$ . It shows that for training ${\hat{B}}_{F,k}$ , information from states, the sliding surface s_k_, the FES input, *u*_*F,k*_, and the value of the neural network weight from the previous time step are used. More mathematical details are provided in Eqs. 18, 19. Additionally, the figure demonstrates the training process for the not linearly parameterizable part of the output dynamics, ${\hat{f}}_{2,k}$ . It shows that the training is done based on information from states, the sliding surface s_k_, and the values of neural network weights from the previous iteration. Mathematical details of training can be found in Eq. 6, (28), (29), and (31). ${\hat{\sigma }}_{k}$ and the neural network weight matrices are saved at the end of each iteration, and training is picked up in the next iteration where it was left off using the saved file that has stored the weight matrices and ${\hat{\sigma }}_{k}$ of the previous iteration. ${\hat{f}}_{2,k}$ is updated after each iteration, and ${\hat{B}}_{F,k}$ is updated at each time instant.

## 3 Experiments

We implemented the proposed controller for repetitive sit-to-stand tasks with a hybrid exoskeleton. The exoskeleton that is shown in Figure 1 was developed in our laboratory (Alibeji et al. (2018b)). The hip joints of the powered exoskeleton are actuated by two LPA-17–100-SP electric motors (Harmonic Drive, United States). These two motors have a maximum speed of 30 revolutions per minute (RPM) and a peak torque of 54 Nm. Two 90-W EC Flat Maxon motors (Maxon Motor, Sachseln, Switzerland) actuate the knee joints of the exoskeleton. The gearbox ratio for both knee and hip joints is 100:1. In the experiments, FES was applied on the quadriceps muscle group for the sit-to-stand task. The NNILC method was used to compute the stabilizing torques for the hip and knee joints during the task, while the MPC method was used to distribute inputs between FES and the electric motors at the knee joints.

The study was approved by the Institutional Review Board (IRB) at the University of Pittsburgh (IRB approval number: PRO 14040419) and the IRB of North Carolina State University (IRB approval number: 20553). Four male participants without any neurological disorders (listed in Table 1) were recruited for the study. Before each experiment, every participant signed an informed consent form.

**TABLE 1:** Anthropometric characteristics of the participants. P1, P2, P3, and P4 represent the first, second, third, and fourth participant.

Participant	Height (cm)	Weight (kg)	Age (years old)
Male 1 (P1)	176.5	70	23
Male 2 (P2)	177.0	78	25
Male 3 (P3)	168.6	65	23
Male 4 (P4)	185.4	74.84	24

Before doing the control validation experiments, a set of trials were conducted on each participant to estimate the knee musculoskeletal model. These experiments were conducted while participants were seated in a leg extension machine. This model identification is needed for the execution of the MPC allocator. The model parameters of the participants, like the fatigue model parameters like the fatigue time constant, $\tau_{f,j}\in R^{+}$ , and the recovery time constant, $\tau_{r,j}\in R^{+}$ , were identified with the procedures reported in our previous works (Kirsch et al. 2018); (Kirsch et al, 2017)). The fatigue and recovery constants for the 4 able-bodied participants on both legs are given in Table 2.

**TABLE 2:** Fatigue and recovery time constants τ_f_ and τ_r_ for participants 1, 2, 3, and 4 on both legs.

			τ_f_ [sec]				
P1 left	P1 right	P2 left	P2 right	P3 left	P3 right	P4 left	P4 right
24.6	23.0	20.2	17.9	25.2	21.6	24.2	26.3
τ_r_ [sec]							
P1 left	P1 right	P2 left	P2 right	P3 left	P3 right	P4 left	P4 right
38.6	47.0	50.8	42.0	43.3	49.1	33.6	29.5

### 3.1 Sit-to-Stand Experiment Protocol

A real-time target machine (Speedgoat, Inc., Liebefeld, Switzerland) running at a control frequency of 400 Hz was used to control the exoskeleton and FES. The control implementation was programmed in Simulink (MathWorks, Inc., United States). The control parameters were programmed based on the following rules: γ = 1, *β*_2_ > 2, and $\alpha_{2}>\frac{2}{\lambda_{2}}$ . These rules were derived using stability analysis provided in Supplementary Appendix B. The main control parameters that were used during experiments are provided in Table 3. A common desired virtual constraint function for the joints of both legs was designed using the methods described in Supplementary Appendix C. The function was designed such that the sit-to-stand task is mainly achieved in 8–11 s. After the transition, the standing position was programmed to be held for up to 15 s. The controllers were implemented separately for each leg, but the controllers used the same virtual constraint function to maintain coordination between the two legs.

**TABLE 3:** Main control parameters that were used during the experiments for all participants.

Parameters	χ	β_1_	β_2_	α_1_	α_2_	q_c_
Value	1	19,231	2.1	0.0769	5,333.3	3
Parameters	λ_1_	λ_2_	γ	ρ_1_	ρ_2_	
Value	0.0375	0.0004	1	2	2	

Stimulation Parameters: A pair of FES electrodes (size: 2 inches × 3.5 inches, Chattanooga Medical Supply, Inc., United States) were placed on the participant’s thighs, after shaving and cleaning the area. The distal electrode was placed on the medial side near the knee joint, while the proximal electrode was placed either at midline or slightly to the lateral side. A biphasic pulse train was delivered to the electrodes using an FES stimulator (RehaStim 8-channel stimulator, Hasomed, Inc., DE). The threshold and saturation current amplitude of the stimulation are defined as the minimal current amplitude that generates observable knee extension torque and the maximal current amplitude that cannot increase knee extension torque, respectively. Both the threshold and saturation current amplitudes were determined by using a set of prior tests (Bao et al. (2020a)). Due to the large current amplitude range between the threshold level and the saturation level (around 50 mA), a current amplitude modulating protocol with a stimulation frequency of 35 Hz and a pulse width of 400 *μs* was chosen in this work.

Since none of the participants had experienced FES or had used an exoskeleton before they were enrolled in the experiments, the participants were trained to properly use the hybrid exoskeleton. During the sit-to-stand task, a walker was used to assist the participant’s balance. There were multiple trials for each participant, and there were four iterations per trial. Between each iteration, we waited for a minute to provide each participant some rest. The trials conducted after the training were chosen for analysis to minimize any unexpected influence of the participants’ unfamiliarity with the hybrid device.

## 3.2 Results

**FIGURE 2:**
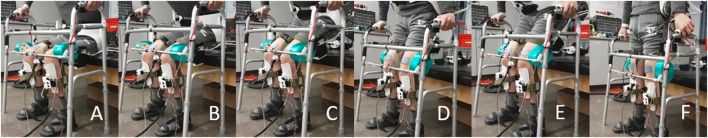
Snapshots of one sit-to-stand trial for Participant 1.

Figure 2 demonstrates the snapshots of the sit-to-stand experiment from one of the trials for Participant 2.

**FIGURE 3:**
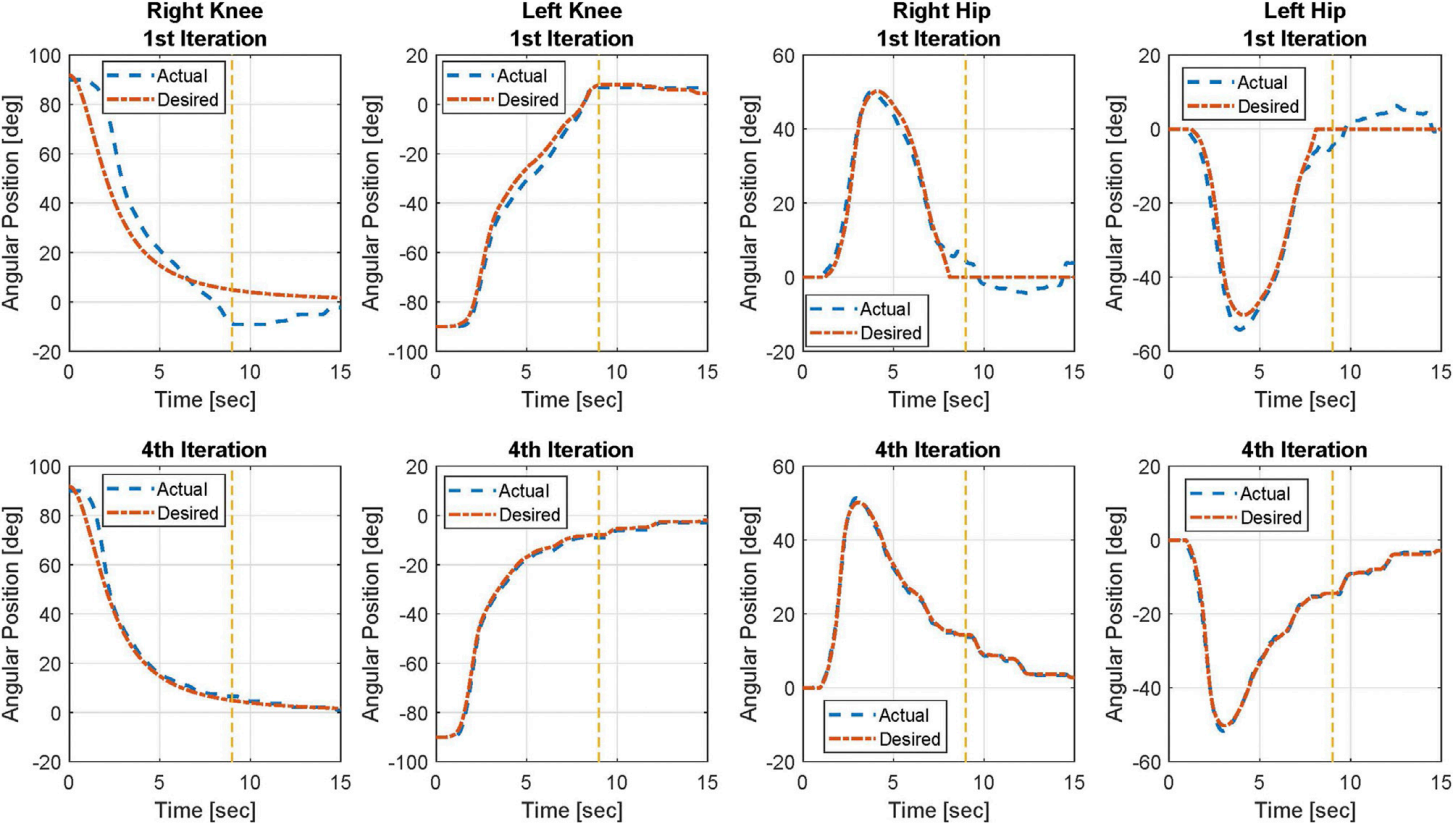
Knee and hip joint angular position tracking results of Participant 2 in the 1st and 4th iterations. Yellow dashed line shows the approximate time in which the sit-to-stand movement is mainly done.

**FIGURE 4:**
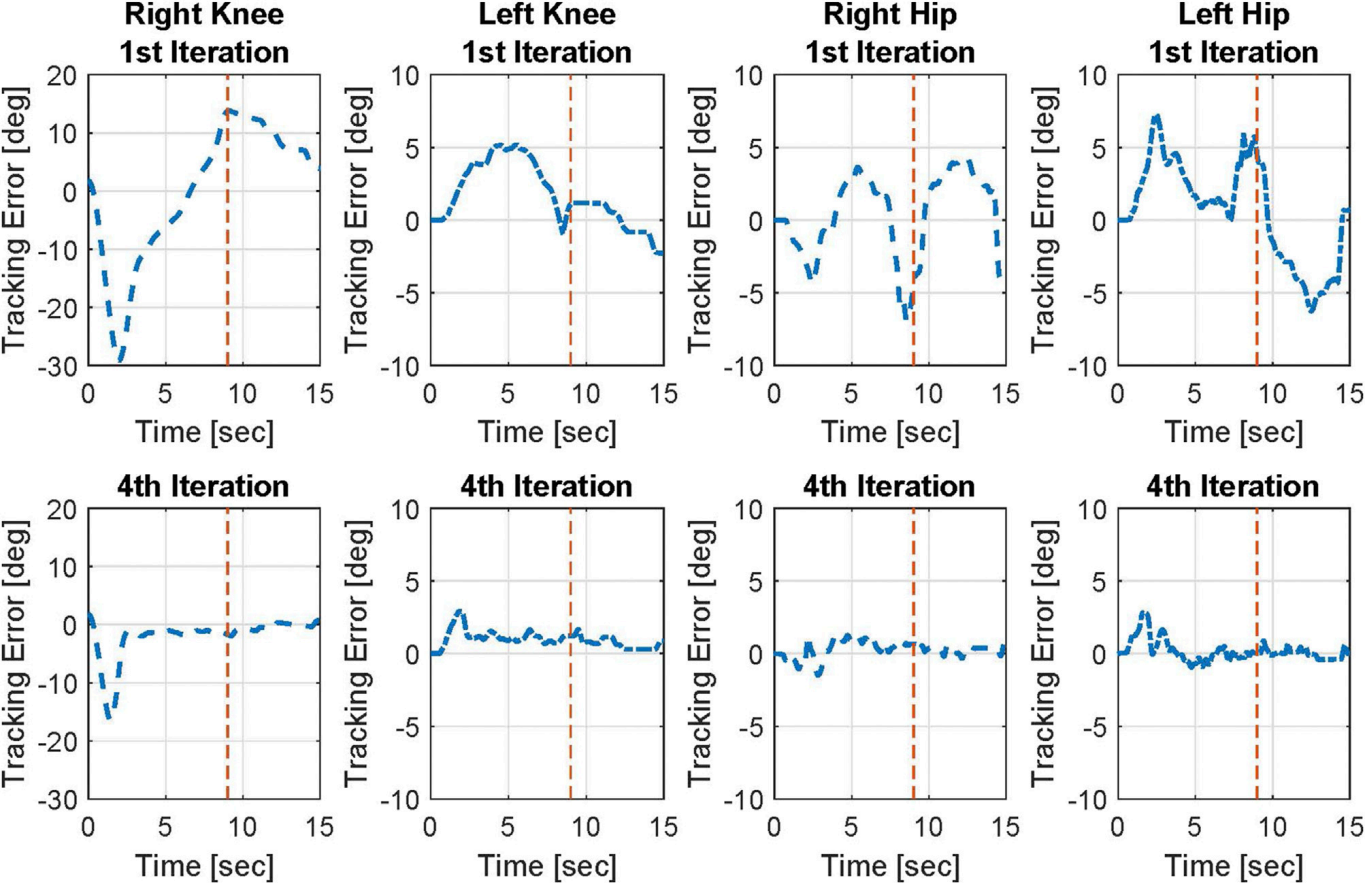
Angular position tracking errors on both knee and hip joints of Participant 2 in the 1st and 4th iterations. Red dashed line shows the approximate time in which the sit-to-stand movement is mainly done.

The trajectory tracking results on both knee and hip joints for Participant 2 are illustrated in Figure 3. The figure includes the desired trajectories that are based on the virtual constraint function and the actual trajectories on both legs in the 1^st^ and 4^th^ iterations. The joint angle tracking errors of Participant 2 in the 1^st^ iteration and the 4^th^ iteration are shown in Figure 4. Left and right legs have different desired profiles. In general, the trajectory tracking errors and the resultant input torques of the left knee joint are lower than those of the right knee joint because the virtual constraints, used as the desired profile for the left knee joint, take the right knee joint actual angular position as the base. However, the desired profile of the right knee joint is pre-designed. Therefore, the right knee joint leads the left knee joint at the initiation of the task and during the movement. Because the right knee joint is the joint that initiates the task, it needs higher torque, and the tracking is more challenging for this joint.

**FIGURE 5:**
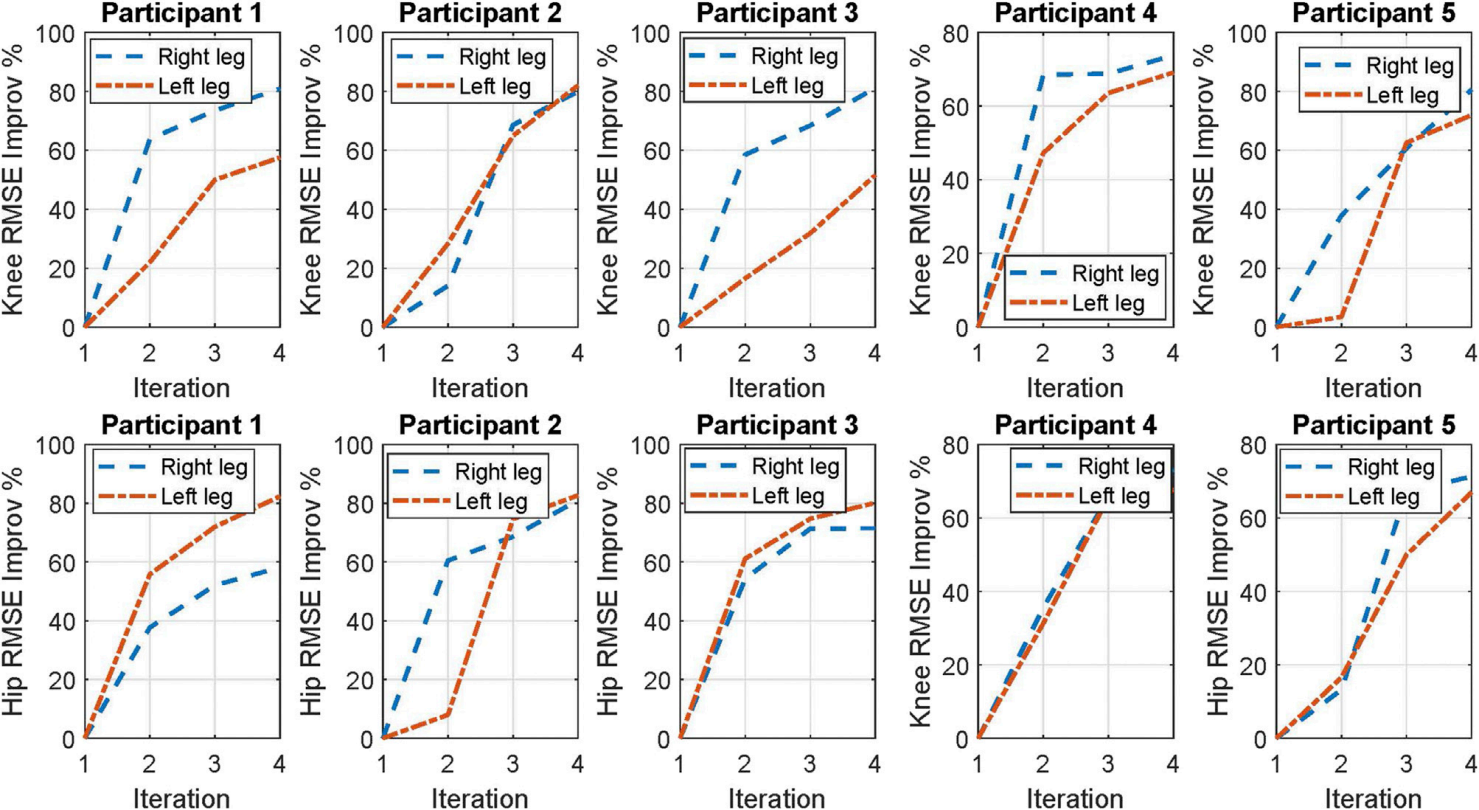
RMSE improvement percentage of both knee and hip joints from the 1st to the 4th iterations for each participant.

The improvement percentage of the RMSE of the joints’ trajectories’ tracking performance for the four participants is plotted in Figure 5. The improvement percentage of the RMSE is calculated based on the following equation:

$${\text{RMSE Improv \%}}_{k}=\left(\frac{RMSE_{1}-RMSE_{k}}{RMSE_{1}}\right)100$$ (20)

The results show that from the 1st iteration to the 4^th^ iteration, for each participant, the RMSE improvement values for both knee and hip joints are increasing. These results indicate that the ILC method improves the joint trajectory tracking performance in successive iterations.

**FIGURE 6:**
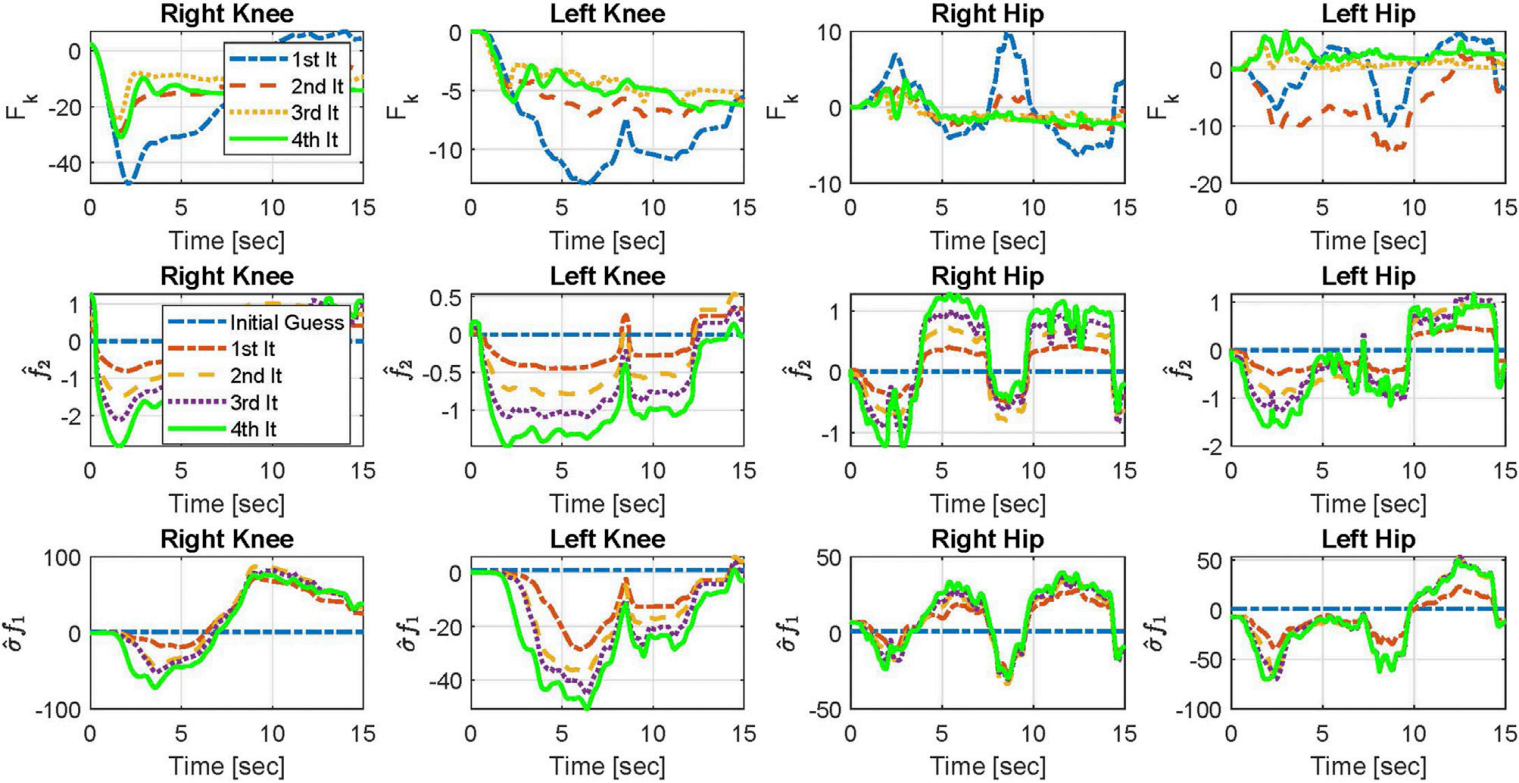
Changes of F_k_, ${\hat{f}}_{2}$ , and $\hat{\sigma }f_{1}$ in the **top-level** controller for Participant 2 in 4 iterations.

In Figure 6, components of the top-level controller, U_k_, in Eq. 4 for Participant 2 in four iterations are shown. The components include F_k_, ${\hat{f}}_{2,k}$ , and ${\hat{\sigma }}_{k}f_{1,k}$ . In this figure, F_k_ represents the additional feedback input and ${\hat{f}}_{2,k}$ , and ${\hat{\sigma }}_{k}f_{1,k}$ represents the not linearly parameterizable and linearly parameterizable elements in the system dynamics learned through iterative fashion. As depicted in this figure, the magnitude of F_k_ decreases while the magnitudes of ${\hat{f}}_{2,k}$ and ${\hat{\sigma }}_{k}f_{1,k}$ increase along with the iterations. Those changes indicate that the contribution of the feedback term F_k_ in the top-level controller is reduced, and the contribution of the learning terms is increased.

**FIGURE 7:**
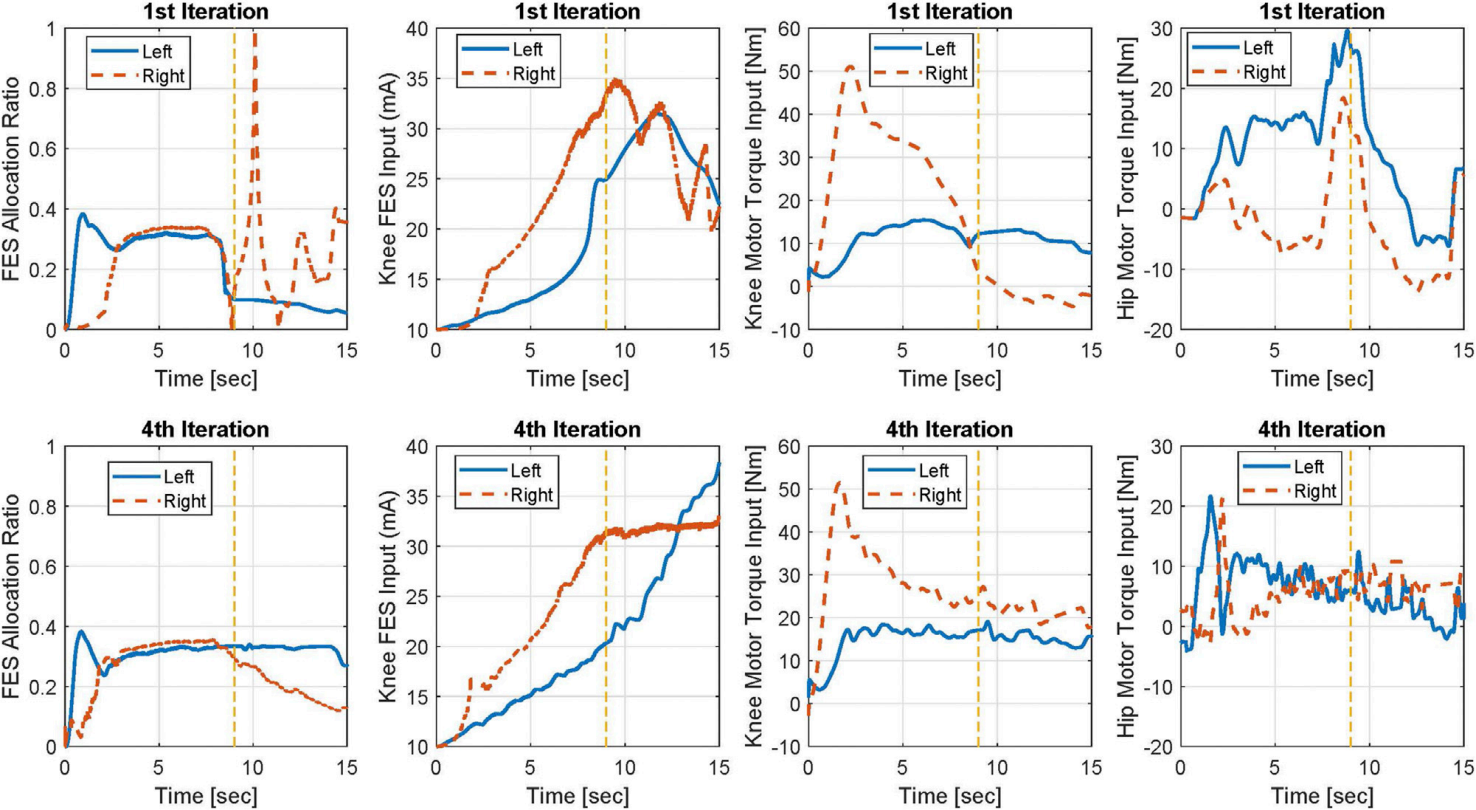
Bottom-level control inputs for Participant 2 allocated by MPC in the 1st and 4th iterations. Yellow dashed line shows the approximate time in which the sit-to-stand movement is mainly done.

The bottom-level control inputs for the 1^st^ and 4^th^ iterations for Participant 2 are shown in Figure 7. In this figure, the allocation ratio for FES is shown in the k^th^ iteration. The allocation ratio shows how much of the top-level control input is allocated to FES by the model predictive allocator. At t = 10 s in the 1st iteration, the knee motor torque magnitude is 0 and the allocation ratio for FES is one.

Detailed experimental results for all 4 participants are provided in Table 4, where ${\overset{{}^{\circ}}{u}}_{M}^{1^{st}It}$ and ${\overset{{}^{\circ}}{u}}_{M}^{4^{th}It}$ show absolute mean values of the motors’ control effort in the 1st and 4th iterations, respectively, ${\overset{{}^{\circ}}{u}}_{F}$ shows the mean value of the normalized FES control effort, and “Improv” stands for improvement.

**TABLE 4:** RMSE of trajectory tracking and inputs from motors and FES on each participant in the 1st and 4th iterations.

	Joints	RMSE 1st iteration	RMSE 4th iteration	RMSE Improv %	${\overset{{}^{\circ}}{u}}_{M}^{1^{st}}$	${\overset{{}^{\circ}}{u}}_{M}^{4^{th}}$	${\overset{{}^{\circ}}{u}}_{F}^{1^{st}}$	${\overset{{}^{\circ}}{u}}_{F}^{4^{th}}$
P1	Right knee	23.11	4.42	80.86	11.28	23.66	0.44	0.41
	Left knee	3.32	1.41	57.52	9.32	13.00	0.26	0.21
	Right hip	1.74	0.72	58.14	6.48	6.64	–	–
	Left hip	4.14	0.73	82.34	7.73	8.89	–	–
P2	Right knee	9.66	1.94	79.91	18.11	26.21	0.38	0.42
	Left knee	3.13	0.56	82.01	11.11	14.86	0.27	0.29
	Right hip	2.04	0.38	81.09	6.41	5.90	–	–
	Left hip	2.56	0.44	82.68	10.93	6.38	–	–
P3	Right knee	23.31	4.39	81.17	12.36	18.89	0.28	0.35
	Left knee	3.33	1.62	51.5	12.33	13.29	0.51	0.25
	Right hip	2.48	0.71	71.45	10.85	5.88	–	–
	Left hip	4.36	0.87	80.15	8.20	7.66	–	–
P4	Right knee	7.28	1.91	73.76	17.24	15.26	0.10	0.003
	Left knee	1.29	0.40	68.99	11.37	10.18	0.11	0.05
	Right hip	5.00	1.34	73.20	8.22	5.23	–	–
	Left hip	4.81	1.56	67.56	8.79	7.06	–	–
All	Mean	6.34	1.46	73.27	10.67	11.81	0.29	0.5
	Std	6.91	1.26	10.08	3.33	6.54	0.15	0.15

**FIGURE 8:**
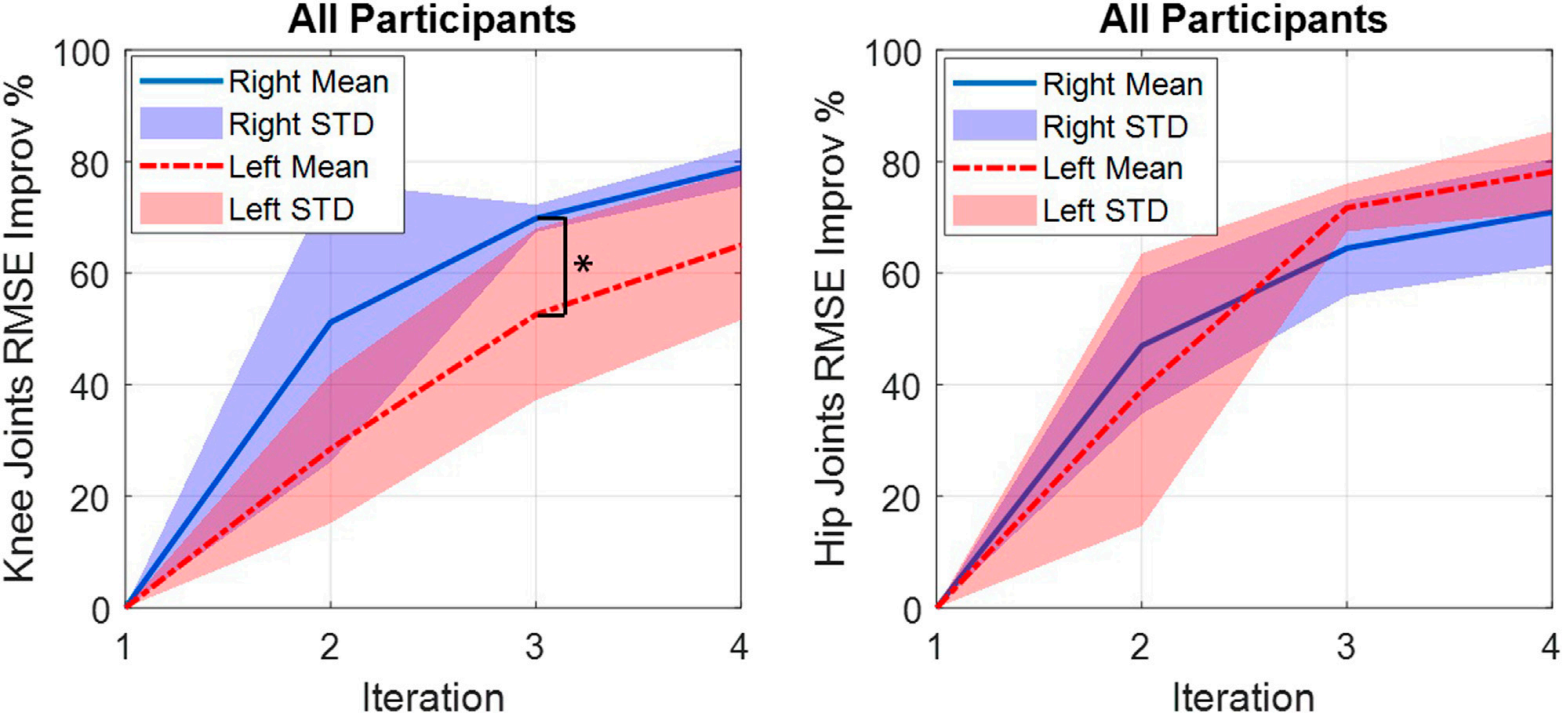
Improvements in the mean RMSE for all participants. Solid and dashed lines show the mean, and the shaded areas show the standard deviation.

According to Figure 8, the novel NNILC method was able to improve the right-knee, left-knee, right-hip, and left-hip RMSEs, 78.92, 65.02, 70.93, and 78.19%, respectively, on average for all participants. For statistical analysis of the novel controller, we focused on the RMSE reduction percentage of each iteration across the four participants and compared the reduction performance between the left knee/hip joint and the right knee/hip joint. For each joint in an individual iteration, there were four RMSE values across participants. A Shapiro–Wilk test was used to determine the normality of the data. The results did not show a normal distribution of RMSE results on each joint. Therefore, a Wilcoxon rank sum test was used to determine if there was a significant difference among the left and right joints’ RMSE reduction percentage in the second, third, and fourth iterations, respectively. We observed significant difference between the left and right knee joints in the third iteration (*p* = 0.029). Other than this, there was no significant difference between the left and right joints in each iteration (detailed *p* values are shown in Figure 8), which indicates a comparative and symmetric performance of the proposed controller on the left and right knee/hip joints.

## 4 Discussion

A hybrid system that combines FES and the powered exoskeleton is a promising rehabilitation intervention to assist people with mobility disorders. Motivated by an ILC approach that improves a system’s transient performance in multiple iterations, the study presented derivation and experimental results of a novel NN-based ILC method. The optimal low-level MPC-based allocator automates the need to specify an allocation ratio between the powered exoskeleton and FES. The allocation process has coupled performance effects. Thus, in a manual tuning involving trial and error, a clinician/physical therapist may lack the relevant control engineering experience to guarantee stability and performance of the system. The proposed bi-level control framework potentially contrasts the manual allocation process, which could be daunting to a clinician/physical therapist. Also, the optimization framework determines optimal allocation ratios instead of static/*ad hoc* nonoptimal allocation, which potentially increases the device efficiency.

The results show that the iterative learning process improves tracking performance by increasing a feed-forward learning part while decreasing the control’s feedback component. The NN-based ILC potentially facilitates the control implementation despite inter-person and day-to-day variations in a user’s FES-driven musculoskeletal dynamics. Most model-based optimal control approaches often involve a tedious process of identifying the model (Stein et al. (1996); Kirsch et al. (2018)), which hinders their control implementation. Instead, nonlinear robust control methods, for example, discontinuous sliding mode control (Bkekri et al. (2018)) and continuous RISE control (Sharma et al. (2009)), have been explicitly designed to address uncertainties in the nonlinear musculoskeletal model. However, these approaches inherently rely on the high frequency or high gain control to compensate for the modeling uncertainties and cause overstimulation. A feed-forward control strategy is usually recommended along with feedback control to reduce overall control effort. Therefore, the FES controllers in the studies by Ajoudani and Erfanian (2009), Lujan and Crago (2009), and Cousin et al. (2019) used neural networks as feed-forward controllers. The NN-based control approach’s advantage is its universal approximation property that helps to capture unstructured uncertainties in the musculoskeletal dynamics (Sharma et al. (2012)). NN-based control, however, requires training to obtain the desired performance. Both offline (Kim et al. (2008); Yu and Rosen (2013)) and online (Sharma et al. (2012)) NN training methods have been used for FES control. In this study, the NN approach uses a combination of online gradient update laws that tune NN weights after every task iteration or at every time instant. A Lyapunov-like stability analysis facilitates the design of these update laws and guarantees the bi-level hierarchical control method’s stability.

Compared to a predetermined higher-level input generator, the NN-based ILC method’s torque generation is more robust to disturbances. Zhang et al. (2015) investigated multiple predetermined higher torque generation techniques based on a limb angle, time, or an EMG signal. A low-level controller then matched the desired torque trajectory. However, the predetermined desired torque may not be robust to perturbations such as spasticity. The robustness to perturbations is essential in situations where the goal is to enable a person with absent motor control to verticalize from sitting. The bi-level control approach used in our study produces robust torque that tracks the desired trajectory. Unlike kinematic tracking, torque-based control of a wearable robot may be more useful in situations where a user may need some torque assistance to control stiffness/impedance or torque. Our focus was on people with completely absent volitional control. Thus, the control design focused on kinematic control instead of torque control.

Repetitive movements such as walking are a good benchmark to test an ILC control method’s performance. For sit-to-stand, each sit-to-stand movement is considered as one iteration, and for the case of walking, each gait cycle is considered as one iteration. We, however, used sit-to-stand experiments to show the feasibility of the NN-based ILC method. Sit-to-stand movement has a very high torque demand, which makes it even more challenging than walking. These tasks are significant as they facilitate a sit-to-stand, a basic movement, a precursor to walking. Also, enabling people with SCI to perform repeated upright standing tasks is beneficial for their musculoskeletal and cardiovascular health. While we do not underestimate the significance of walking, which is critical to mobility and is our ultimate goal, we emphasize sit-to-stand as an equally challenging control problem and actively pursued research on it, for example, in the studies by Alouane et al. (2019), Huo et al. (2016), and Jatsun et al. (2015).

In this work, state-dependent manifolds are used as reference trajectories. This desired trajectory design approach differs from our previous work in the study by Alibeji et al. (conditionally accepted, 2018a) that used time-dependent reference trajectories. Some SCI participants may exhibit asymmetric left and right leg movements during the sit-to-stand task. The time-dependent desired trajectories in this situation may not correct themselves and may produce an uncoordinated movement, potentially uncomfortable and unsafe for a user. In our current approach, the joint reference profiles adapt based on the current state of the system. The state-dependent desired trajectory design coordinates both legs’ hip and knee joints, enabling the users to achieve a more stable and natural movement.

Two limitations in the study deserve discussion. First, we could have used pulse width (PW) modulation, instead of current amplitude (CA) modulation, for FES control. Although both PW and CA modulation have the same function of increasing and decreasing muscle fiber recruitment, the stimulator (Rehastim, Hasomed, Inc.) used in the study has a higher resolution for PW modulation than the current modulation. We will be switching our future work to the PW mode.

Furthermore, the results from subjects with no disability verified the proposed bi-level NNILC-MPC framework’s feasibility to optimally allocate FES and the powered exoskeleton. The experimental results on the participants with no disabilities here are preliminary and more experiments on participants with SCI will be performed to validate the benefits of the control framework further.

## 5 Conclusion

A novel NNILC augmented with an MPC-based allocation strategy was developed to control a hybrid exoskeleton in this work. A Lyapunov-like stability analysis proved that the unified control framework yielded asymptotic tracking performance despite uncertain dynamics and disturbances. State-dependent trajectories were used as desired joint trajectories. The experimental results of four participants without a disability demonstrated that the controller enabled sit-to-stand tasks. The tracking performance showed improvement in each iteration. The results also showed that the MPC strategy could achieve the optimal allocation between FES and the powered exoskeleton.

## Data Availability

The raw data supporting the conclusions of this article will be made available by the authors, without undue reservation.
