# Supplementary material for: Shared Control of a Powered Exoskeleton and Functional Electrical Stimulation Using Iterative Learning
Source: Front Robot AI. 2021 Nov 3;8:711388. doi: 10.3389/frobt.2021.711388 (PMC8595125; doi:10.3389/frobt.2021.711388)
Supplement: Supplementary file 1 [file DataSheet1.pdf]

frontiers

Supplementary File for Shared Control of a Powered Exoskeleton and Functional Electrical Stimulation using Iterative Learning and Fatigue Optimization

Vahidreza Molazadeh [1], Qiang Zhang [2], Xuefeng Bao [3], Brad E. Dicianno [4] and Nitin Sharma [2,*]

[1] *Department of Mechanical Engineering and Material Science, University of Pittsburgh, Pittsburgh , PA, USA*
[2] *Neuromuscular Control and Robotics lab, Joint Department of Biomedical Engineering, North Carolina State University and the University of North Carolina Chapel-Hill, Raleigh , NC, USA*
[3] *Department of Biomedical Engineering at University of Wisconsin-Milwaukee, Milwaukee, Wisconsin, USA*
[4] *Department of Physical Medicine and Rehabilitation, School of Medicine and Department of Bioengineering, University of Pittsburgh, Pittsburgh, PA, USA*

Correspondence*:
Nitin Sharma
nsharm23@ncsu.edu

APPENDIX A. CONTROL DEVELOPMENT

For subsequent control development, the model in (1) is rewritten as

$$\dot{\boldsymbol{x}} = \boldsymbol{f}(\boldsymbol{x}) + \boldsymbol{g}_F(\boldsymbol{x})\boldsymbol{u}_F + \boldsymbol{g}_M\boldsymbol{u}_M + \boldsymbol{D}_x \tag{21}$$

where $\boldsymbol{x} \in \mathbb{R}^{2N}$ is defined as $\boldsymbol{x} = \left[\boldsymbol{\theta}^T, \dot{\boldsymbol{\theta}}^T\right]^T$, $\boldsymbol{f}(\boldsymbol{x}) \in \mathbb{R}^{2N}$ is defined as $\boldsymbol{f}(\boldsymbol{x}) = \left[\dot{\boldsymbol{\theta}}^T, \left[\boldsymbol{M}^{-1}(-\boldsymbol{T}_p - \boldsymbol{G} - \boldsymbol{C}\dot{\boldsymbol{\theta}})\right]^T\right]^T$, $\boldsymbol{g}_F \in \mathbb{R}^{2N\times N_f}$ is FES gain matrix, and $\boldsymbol{g}_M \in \mathbb{R}^{2N\times N_m}$ is motor gain matrix and $\boldsymbol{D}_x \in \mathbb{R}^{2N}$ is defined as $\boldsymbol{D}_x = \left[\boldsymbol{\emptyset}_{1\times N}, \left[\boldsymbol{M}^{-1}\boldsymbol{D}\right]^T\right]^T$.

Output error dynamics

$\boldsymbol{\theta}$ is the actual joint angle vector in (1). The control objective is to ensure that the independent joint angle function, $\boldsymbol{\theta} \in \mathbb{R}^N$, follows a desired virtual constraint function (Westervelt et al. (2007)), $\boldsymbol{h}(\boldsymbol{\theta}) \in \mathbb{R}^N$,. Note that the virtual constraint function is a function of system state instead of an explicit function of time. The reason for designing desired trajectory in this way is to avoid joints miscoordination that can be caused by totally independent time based desired trajectories. The method to design the desired virtual constraint function is given in Appendix C. The control objective can be expressed as an output, $\boldsymbol{y} \in \mathbb{R}^N$, that must be driven to zero. Thus, the output, $\boldsymbol{y}$, is defined as

$$\boldsymbol{y} \triangleq \boldsymbol{\theta} - \boldsymbol{h}. \tag{22}$$

Using (22), the following output differential equation is derived

$$\frac{d^2\boldsymbol{y}}{dt^2} = \boldsymbol{L}_f^2\boldsymbol{y} + \boldsymbol{L}_{\boldsymbol{g}_M}\boldsymbol{L}_f\boldsymbol{y}\boldsymbol{u}_{\overline{M}} + \boldsymbol{L}_{\boldsymbol{g}_F}\boldsymbol{L}_f\boldsymbol{y}\boldsymbol{u}_{\overline{F}} + \boldsymbol{d} \tag{23}$$

where $\boldsymbol{L}_f^2\boldsymbol{y}$ is the 2nd-order Lie derivative of $\boldsymbol{y}$, $\boldsymbol{L}_{\boldsymbol{g}_M}\boldsymbol{L}_f\boldsymbol{y}$ and $\boldsymbol{L}_{\boldsymbol{g}_F}\boldsymbol{L}_f\boldsymbol{y}$ are the decoupling matrices, and $\boldsymbol{d} \in \mathbb{R}^N$ is the disturbance of the system output.

We consider that $\bar{y}_1^{(i)} = y^{(i)}$, $\bar{y}_2^{(i)} = \dot{y}^{(i)}$, where superscript $(i) = 1, 2, ..., N$ shows i^{th} row of a vector. As the ultimate motivation is to develop a novel Iterative learning control (ILC) approach, (23) is expressed for a kth iteration as

$$\begin{aligned} \dot{\bar{y}}_{1,k}^{(i)} &= \bar{y}_{2,k}^{(i)} \\ \dot{\bar{y}}_{2,k}^{(i)} &= (\sigma f_{1,k})^{(i)} + f_{2,k}^{(i)} + \left(\sum_{j=1}^{N_f} B_{F,j,k}u_{F,j,k}\right)^{(i)} + \left(\sum_{o=1}^{N_m} B_{M,o}u_{M,o,k}\right)^{(i)} + d_k^{(i)} \end{aligned} \tag{24}$$

where $\left(\sigma f_{1,k} + f_{2,k}\right)^{(i)}$ is equal to i^{th} element of $\boldsymbol{L}_f^2\boldsymbol{y}$ and is expressed as a sum of structured and unstructured uncertain nonlinear terms. Specifically, $(\sigma f_{1,k})^{(i)}$ is the linearly parameterizable (structured uncertainty) part of ith element of $\boldsymbol{L}_f^2\boldsymbol{y}$, where $\sigma^{(i)}$ is an unknown parameter function and $f_{1,k}^{(i)}$ is a known regressor function, and $f_{2,k}^{(i)}$ is the remaining not linearly parameterizable (unstructured uncertainty) part of ith element of $\boldsymbol{L}_f^2\boldsymbol{y}$. In (24), $B_{M,o}$ and $B_{F,k}$ are equal to $\boldsymbol{L}_{\boldsymbol{g}_M}\boldsymbol{L}_f\boldsymbol{y}$ and $\boldsymbol{L}_{\boldsymbol{g}_F}\boldsymbol{L}_f\boldsymbol{y}$, respectively. Here, $B_{M,o}$ is the motor control constant gain for o^{th} electric motor input. $u_{M,o}$ and is assumed to be known. $B_{F,j,k}$ is an unknown control gain associated with j^{th} normalized FES input, $u_{F,j,k}$. Normalized FES input, $u_{F,j,k}$ is bounded as follow

$$|u_{F,j,k}| \leq 1 \tag{25}$$

N_F is the total number of electrical stimulator and N_m is the total number of motors. For simplicity purposes, the superscript i $(i = 1,\ 2,\ ...,\ N)$ will be dropped hereafter in all the corresponding notations. We assume that the terms $f_{2,k}$ and $B_{F,k}$ can be represented using two ideal NNs as follows

$$f_{2,k} = \boldsymbol{W}^T\boldsymbol{\Lambda}_k(\boldsymbol{P}^T\boldsymbol{X}_k) + \varepsilon_{1,k}\left(\boldsymbol{X}_k\right) \tag{26}$$

$$B_{F,j,k} = \boldsymbol{Q_j}^T\boldsymbol{\phi}_{j,k}(\boldsymbol{X}_k) + \varepsilon_{2,j,k}\left(\boldsymbol{X}_k\right) \tag{27}$$

where $\boldsymbol{X}_k \in \mathbb{R}^{2N+1}$ is the augmented input vector for the aforementioned two NNs and is defined as $\boldsymbol{X}_k = \begin{bmatrix} 1 & \boldsymbol{x}_k^T \end{bmatrix}^T$. $\boldsymbol{W} \in \mathbb{R}^{N_2+1\times N}$ and $\boldsymbol{P} \in \mathbb{R}^{2N+1\times N_{in}}$ are the ideal weight matrices for term $f_{2,k}$ and $\boldsymbol{Q} \in \mathbb{R}^{N_\Omega\times N}$ is the ideal weight vector for $B_{F,k}$. The input layer is made of $2N+1$ neurons. N is the output layer neurons number, and the hidden layer numbers of neurons in the two NNs are N_{in} and N_Ω. $\boldsymbol{\Lambda}_k : \mathbb{R}^{N_{in}} \to \mathbb{R}^{N_2+1}$ is the first NN activation function in (26) that maps the input layer to the hidden layer, where $\boldsymbol{P} \in \mathbb{R}^{(2N+1)\times N_{in}}$ is the weight matrix corresponding to the augmented input. $\boldsymbol{\phi}_{j,k} : \mathbb{R}^{2N+1} \to \mathbb{R}^{N_\Omega}$ is the activation function in (27) that maps the input layer to the output layer. $\varepsilon_{1,k} \in \mathbb{R}^{\mathrm{N}}$ and $\varepsilon_{2,k} \in \mathbb{R}^{\mathrm{N}}$ are the unknown functional reconstruction errors for the two NNs, which all elements of them are bounded with $\left|\varepsilon_{1,j,k}\right| \leq \bar{\varepsilon}_1$ and $\left|\varepsilon_{2,j,k}\right| \leq \bar{\varepsilon}_2$, respectively, where $\bar{\varepsilon}_1,\ \bar{\varepsilon}_2 \in \mathbb{R}^+$ (Chen

et al. (2010)). By the NN universal approximation property the bounds on the functional reconstruction errors can be made smaller by choosing a larger number of neurons in the layers (Lewis et al. (2002)).

Based on the subsequent stability analysis, the estimates of ideal weight matrices in the NNS, $\hat{\boldsymbol{W}}_{k_j}$, $\hat{\boldsymbol{P}}_{k_j}$, and $\hat{\boldsymbol{Q}}_{j,k}$ are updated using the following gradient method

$$\hat{\boldsymbol{W}}_{k_j} = \hat{\boldsymbol{W}}_{(k-1)_j} - \rho_1 \frac{\partial E_{f_{2,k}}}{\partial \hat{\boldsymbol{W}}_{(k-1)_j}} \tag{28}$$

$$\hat{\boldsymbol{P}}_{k_j} = \hat{\boldsymbol{P}}_{(k-1)_j} - \rho_2 \frac{\partial E_{f_{2,k}}}{\partial \hat{\boldsymbol{P}}_{(k-1)_j}} \tag{29}$$

$$\dot{\hat{\boldsymbol{Q}}}_{j,k} = -\chi \boldsymbol{\phi}_{j,k} \left(\boldsymbol{X}_k \right) u_{F,j,k} s_k. \tag{30}$$

where $\rho_1 \in \mathbb{R}^+$ and $\rho_2 \in \mathbb{R}^+$ are user defined constants, j represents j^{th} element of a matrix and $E_{f_{2,k}}$ is defined as

$$\begin{array}{c} E_{f_{2,k}} = \frac{1}{2} \left(\hat{f}_{2,k} - \hat{f}_{2,k-1} + \xi \gamma s_k \right)^2 \\ f_{2,k} = 0, \text{ when } k = -1 \end{array} . \tag{31}$$

Closed-loop error dynamics

Using (17), by adding and subtracting $\sum_{j=1}^{N_f} \psi_{j,k} u_{F,j,k}$ to (24) results in

$$\begin{array}{c} \dot{y}_{2,k} = f_{2,k} + \sigma f_{1,k} - \sum_{j=1}^{N_f} \psi_{j,k} u_{F,j,k} \\ + \sum_{j=1}^{N_f} \left(\tilde{\boldsymbol{Q}}_{j,k}^T \boldsymbol{\phi}_{j,k} \left(\boldsymbol{X}_k \right) + \beta_{\varepsilon,j} \right) u_{F,j,k} + \sum_{o=1}^{N_m} b_{M,o} u_{M,o,k} + d_k \end{array} \tag{32}$$

where $\tilde{\boldsymbol{Q}}_{j,k} = \boldsymbol{Q_j} - \hat{\boldsymbol{Q}}_{j,k}$ is the weight estimation error, and $\beta_{\varepsilon,j} = \varepsilon_{2,j,k} - (\varrho_j)$ and it is bounded by $\bar{\beta}_{\varepsilon,j} \in \mathbb{R}^+$. (32) describes the closed loop dynamic of the output. It shows how the stimulation amplitude level and motor torques are contributing to the output dynamics. Additionally, it shows how the estimation errors for control gain matrix and the system dynamics can have influence on the behavior of the output of the closed loop system. Therefore, by substituting (7) in (4) and considering (11) , the following equation is resulted based on (32)

$$\begin{array}{c} \dot{y}_{2,k} = \sigma f_{1,k} + f_{2,k} + d_k + \Big(-\hat{f}_{2,k} - \hat{\sigma}_k f_{1,k} \\ -\frac{\lambda_1}{\lambda_2} \bar{y}_{2,k} + \\ + \left(\alpha_2 s_k + \frac{4}{3} \alpha_1 \text{sgn}(s_k) - I_k \right) \Big) \\ + \sum_{j=1}^{N_f} \left(\tilde{\boldsymbol{Q}}_{j,k}^T \boldsymbol{\phi}_{j,k} \left(\boldsymbol{X}_k \right) + \beta_{\varepsilon,j} \right) u_{F,j,k} \end{array} . \tag{33}$$

Using (33) and (3), the dynamics of the sliding surface can be obtained that

$$\begin{aligned} \dot{s}_k &= -\lambda_1 \bar{y}_{2,k} + \lambda_2 \Big(-\sigma f_{1,k} - f_{2,k} - d_k \\ &- \sum_{j=1}^{N_f} \left(\widetilde{\boldsymbol{Q}}_{j,k}^T \boldsymbol{\phi}_{j,k} \left(\boldsymbol{X}_k \right) + \beta_{\varepsilon,j} \right) u_{F,j,k} + \Big(-\tfrac{\lambda_1}{\lambda_2} \bar{y}_{1,d,k} \\ &+ \tfrac{\lambda_1}{\lambda_2} \bar{y}_{2,k} - \left(\alpha_2 s_k + \tfrac{4}{3} \alpha_1 \mathrm{sgn}(s_k) - I_k \right) + \hat{f}_{2,k} + \hat{\sigma}_k f_{1,k} \Big) \Big) \end{aligned} \quad . \tag{34}$$

Based on (34), the sliding surface's dynamic equation can be rewritten as

$$\begin{aligned} \dot{s}_k &= \lambda_2 \Big(-\alpha_2 s_k - \tfrac{4}{3} \alpha_1 \mathrm{sgn}(s_k) + I_k - d_k \\ &- \widetilde{\sigma}_k f_{1,k} - \sum_{j=1}^{N_f} \beta_{\varepsilon,j} u_{F,j,k} - \sum_{j=1}^{N_f} \widetilde{\boldsymbol{Q}}_{j,k}^T \boldsymbol{\phi}_{j,k} \left(\boldsymbol{X}_k \right) u_{F,j,k} - \widetilde{f}_{2,k} \Big) \end{aligned} \tag{35}$$

where $\widetilde{\sigma}_k = \sigma - \hat{\sigma}_k$, and $\widetilde{f}_{2,k} = f_{2,k} - \hat{f}_{2,k}$.

APPENDIX B: STABILITY ANALYSIS

THEOREM 1. *∀ i=1,...,N, the subsystem in (24) can reach the equilibrium point* $(s_i, e_{0,i}) = (0, 0)$ *asymptotically, if the control inputs are selected as (4) and (16).*

The following Lyapunov-like functional is considered

$$V_k = V_k^{(1)} + V_k^{(2)} + V_k^{(3)} + V_k^{(4)} + V_k^{(5)} \tag{36}$$

where $V_k^{(1)} = \frac{I_k^2}{2\beta_1}$, $V_k^{(2)} = \frac{\gamma}{\lambda_2} \frac{s_k^2}{2}$, $V_k^{(3)} = \frac{1}{2q_c} \int_{t_0}^{t} \widetilde{\sigma}_k^2 d\tau$, $V_k^{(4)} = \frac{1}{2\xi} \int_{t_0}^{t} \widetilde{f}_{2,k}^2 d\tau$, and $V_k^{(5)} = \frac{1}{2\chi} \sum_{j=1}^{N_f} tr \left\{ \tilde{\boldsymbol{Q}}_{j,k}^T \tilde{\boldsymbol{Q}}_{j,k} \right\}$. t_0 is the start time of iterations, t is the elapsed time after the start of an iteration and γ, q_c, $\xi \in \mathbb{R}^+$ are constants. It is considered that at the beginning of each iteration, the exoskeleton wearer has same sitting initial position. Subsequent mathematical procedure proves that the tracking error and estimation error converge to zero based on the energy difference between two successive iterations. Accordingly, for $V_k^{(1)}$, the energy difference between each two iterations can be written as

$$\Delta V_k^{(1)} = V_k^{(1)} - V_{k-1}^{(1)}. \tag{37}$$

Then the following formula can be obtained

$$\Delta V_k^{(1)} = \frac{I_k^2}{2\beta_1} - \frac{I_{k-1}^2}{2\beta_1} = \frac{1}{\beta_1} \int_{t_0}^{t} I_k \dot{I}_k d\tau + \frac{I_k^2(t_0)}{2\beta_1} - \frac{I_{k-1}^2}{2\beta_1} \quad . \tag{38}$$

The following equation is derived by substituting (8) to equation (38)

$$\begin{aligned} \Delta V_k^{(1)} &= -\int_{t_0}^{t} I_k s_k d\tau - \frac{\beta_2}{\beta_1} \int_{t_0}^{t} I_k^2 d\tau \\ &+ \frac{I_k^2(t_0)}{2\beta_1} - \frac{I_{k-1}^2}{2\beta_1}. \end{aligned} \tag{39}$$

The difference of the second energy function for k^{th} and $(k-1)^{th}$ iterations are obtained as

$$\begin{array}{c} \Delta V_k^{(2)} = \frac{\gamma}{\lambda_2}\frac{s_k^2}{2} - \frac{\gamma}{\lambda_2}\frac{s_{k-1}^2}{2} \\ = \frac{\gamma}{\lambda_2}\int\limits_{t_0}^{t} s_k \dot{s}_k d\tau + \frac{\gamma}{\lambda_2}\frac{s_k^2(t_0)}{2} - \frac{\gamma}{\lambda_2}\frac{s_{k-1}^2}{2} \end{array} . \tag{40}$$

By considering the upper bounds, $\bar{d}$ for d_k, $\bar{\beta}_\varepsilon$ for β_ε, considering (25), and substituting (35) to (40), the following equation is achieved

$$\begin{array}{c} \Delta V_k^{(2)} \le -\frac{\gamma}{\lambda_2}\frac{s_{k-1}^2}{2} - \gamma\alpha_2 \int\limits_{t_0}^{t} s_k^2 d\tau + \frac{\gamma}{\lambda_2}\frac{s_k^2(t_0)}{2} \\ +\bar{d}\gamma \int\limits_{t_0}^{t} |s_k|\, d\tau + \gamma \int\limits_{t_0}^{t} s_k I_k d\tau + \gamma \sum\limits_{j=1}^{N_f} \bar{\beta}_{\varepsilon,j} \int\limits_{t_0}^{t} |s_k|\, d\tau \\ -\gamma \int\limits_{t_0}^{t} s_k \widetilde{\sigma}_k f_{1,k} d\tau + \frac{4}{3}\alpha_1\gamma \int\limits_{t_0}^{t} |s_k|\, d\tau \\ -\gamma \int\limits_{t_0}^{t} s_k \widetilde{f}_{2,k} d\tau - \gamma \sum\limits_{j=1}^{N_f}\int\limits_{t_0}^{t} s_k \widetilde{\boldsymbol{Q}}_{j,k}^T \boldsymbol{\phi}_{j,k}\left(\boldsymbol{X}_k\right) u_{F,j,k} d\tau \end{array} . \tag{41}$$

The third energy function difference between two successive iterations can be obtained as

$$\Delta V_k^{(3)} = \frac{1}{2q_c}\int\limits_{t_0}^{t} \widetilde{\sigma}_k^2 d\tau - \frac{1}{2q_c}\int\limits_{t_0}^{t} \widetilde{\sigma}_{k-1}^2 d\tau \ . \tag{42}$$

Based on the theorem in Chen et al. (2012), the following equation can be derived

$$\begin{array}{c} \frac{1}{2q_c}\left(\widetilde{\sigma}_k^2 - \widetilde{\sigma}_{k-1}^2\right) \\ = \frac{1}{2q_c}\left(\hat{\sigma}_k - \hat{\sigma}_{k-1}\right)\left(\hat{\sigma}_k + \hat{\sigma}_{k-1} - 2\sigma\right) \\ = \frac{1}{q_c}\left(\hat{\sigma}_k - \sigma\right)\left(\hat{\sigma}_k - \hat{\sigma}_{k-1}\right) \\ -\frac{1}{2q_c}\left(\hat{\sigma}_k - \hat{\sigma}_{k-1}\right)^2 \end{array} . \tag{43}$$

By substituting (5) to (43) and substituting the results to (42), the following expression can be obtained

$$\begin{array}{c} \Delta V_k^{(3)} = -\frac{1}{2q_c}\int\limits_{t_0}^{t} \left(\hat{\sigma}_k - \hat{\sigma}_{k-1}\right)^2 d\tau \\ -\gamma \int\limits_{t_0}^{t} \left(\widetilde{\sigma}_k s_k f_{1,k}\right) d\tau \end{array} . \tag{44}$$

The fourth energy function difference between two iterations is given as

$$\Delta V_k^{(4)} = \frac{1}{2\xi}\int\limits_{t_0}^{t} \widetilde{f}_{2,k}^2 d\tau - \frac{1}{2\xi}\int\limits_{t_0}^{t} \widetilde{f}_{2,k-1}^2 d\tau. \tag{45}$$

Similarly, it can be rewritten as

$$\begin{array}{c}\frac{1}{2\xi}\left(\widetilde{f}_{2,k}^{2}-\widetilde{f}_{2,k-1}^{2}\right)=-\frac{1}{2\xi}\left(\hat{f}_{2,k}-\hat{f}_{2,k-1}\right)^{2}\\ +\frac{1}{\xi}\left(\hat{f}_{2,k}-f_{2,k}\right)\left(\hat{f}_{2,k}-\hat{f}_{2,k-1}\right)\end{array}. \tag{46}$$

By considering the update laws in (28),(29), and (31), the fourth energy function difference will be given as follows

$$\begin{array}{c}\Delta V_{k}^{(4)}=-\frac{1}{2\xi}\int\limits_{t_0}^{t}\left(\hat{f}_{2,k}-\hat{f}_{2,k-1}\right)^{2}d\tau\\ -\gamma\int\limits_{t_0}^{t}\left(\widetilde{f}_{2,k}s_k\right)d\tau\end{array}. \tag{47}$$

The fifth energy function difference between two iterations can be written as

$$\Delta V_{k}^{(5)}=\sum_{j=1}^{N_f}\left(\frac{1}{2\chi}tr\left\{\widetilde{\boldsymbol{Q}}_{j,k}^{T}\widetilde{\boldsymbol{Q}}_{j,k}\right\}-\frac{1}{2\chi}tr\left\{\widetilde{\boldsymbol{Q}}_{j,k-1}^{T}\widetilde{\boldsymbol{Q}}_{j,k-1}\right\}\right). \tag{48}$$

By considering $\widetilde{\boldsymbol{Q}}_{j,k}(t_0)=0$, $\Delta V_k^{(5)}$ can be written as

$$\Delta V_{k}^{(5)}=\sum_{j=1}^{N_f}\left(-\frac{1}{\chi}tr\left\{\int\limits_{t_0}^{t}\widetilde{\boldsymbol{Q}}_{j,k}^{T}\dot{\widetilde{\boldsymbol{Q}}}_{j,k}d\tau\right\}-\frac{1}{2\chi}tr\left\{\widetilde{\boldsymbol{Q}}_{j,k-1}^{T}\widetilde{\boldsymbol{Q}}_{j,k-1}\right\}\right). \tag{49}$$

By substituting (19) in (49), $\Delta V_k^{(5)}$ becomes

$$\begin{array}{c}\Delta V_{k}^{(5)}=-\frac{1}{2\chi}\sum\limits_{j=1}^{N_f}tr\left\{\widetilde{\boldsymbol{Q}}_{j,k-1}^{T}\widetilde{\boldsymbol{Q}}_{j,k-1}\right\}\\ +\sum\limits_{j=1}^{N_f}tr\left\{\int\limits_{t_0}^{t}\widetilde{\boldsymbol{Q}}_{j,k}^{T}\left(\boldsymbol{\phi}_{j,k}\left(\boldsymbol{X}_k\right)u_{F,j,k}s_k\right)d\tau\right\}\end{array}. \tag{50}$$

To show the convergence of the output tracking error and parameters estimation error, based on the fact that $s_k\in\mathbb{R}$, define $\gamma=1$, and $\alpha_1=\frac{-3(\bar{d}+\bar{\varepsilon}_1+\sum_{j=1}^{N_f}\bar{\beta}_{\varepsilon,j})}{4}$ then combine the aforementioned five energy

difference terms for the two successive iterations, finally, the following ultimate inequality can be resulted

$$\begin{array}{c} \Delta V_k = \Delta V_k^1 + \Delta V_k^2 + \Delta V_k^3 + \Delta V_k^4 + \Delta V_k^5 \\ \leq -\frac{1}{2\xi} \int\limits_{t_0}^{t} \left(\hat{f}_{2,k} - \hat{f}_{2,k-1} \right)^2 d\tau \\ + \frac{\gamma}{\lambda_2} \frac{s_k^2(t_0)}{2} - \gamma\alpha_2 \int\limits_{t_0}^{t} s_k^2 d\tau - \frac{\gamma}{\lambda_2} \frac{s_{k-1}^2}{2} \\ + \frac{I_k^2(t_0)}{2\beta_1} - \frac{\beta_2}{\beta_1} \int\limits_{t_0}^{t} I_k^2 d\tau - \frac{I_{k-1}^2}{2\beta_1} \\ -\frac{1}{2q_c} \int\limits_{t_0}^{t} \left(\hat{\sigma}_k - \hat{\sigma}_{k-1} \right)^2 d\tau - \frac{1}{2\chi} \sum\limits_{j=1}^{N_f} tr \left\{ \widetilde{\boldsymbol{Q}}_{j,k-1}^T \widetilde{\boldsymbol{Q}}_{j,k-1} \right\} \end{array}. \tag{51}$$

By selecting $\beta_2 > 2$ and $\alpha_2 > \frac{2}{\lambda_2}$, (51) can be more simplified as follow

$$\begin{array}{c} \Delta V_k \leq -\alpha_4 \int\limits_{t_0}^{t} I_k^2 d\tau - \frac{I_{k-1}^2}{2} - \alpha_3 \int\limits_{t_0}^{t} s_k^2 d\tau - \frac{\gamma}{\lambda_2} \frac{s_{k-1}^2}{2} \\ -\frac{1}{2\chi} \sum\limits_{j=1}^{N_f} tr \left\{ \widetilde{\boldsymbol{Q}}_{j,k-1}^T \widetilde{\boldsymbol{Q}}_{j,k-1} \right\} - \frac{1}{2q_c} \int\limits_{t_0}^{t} \left(\hat{\sigma}_k - \hat{\sigma}_{k-1} \right)^2 d\tau \\ -\frac{1}{2\xi} \int\limits_{t_0}^{t} \left(\hat{f}_{2,k} - \hat{f}_{2,k-1} \right)^2 d\tau \end{array} \tag{52}$$

where $\alpha_3 \in \mathbb{R}^+$ and $\alpha_4 \in \mathbb{R}^+$. Therefore, it can be concluded that

$$\Delta V_k \leq -\kappa(s_k,\, s_{k-1},\, I_k,\, I_{k-1},\, \hat{f}_{2,k},\, \hat{f}_{2,k-1},\, \hat{\sigma}_k,\, \hat{\sigma}_{k-1},\, \widetilde{\boldsymbol{Q}}_{j,k-1}). \tag{53}$$

Because κ is a class K function, V_k is monotonically decreasing. In order to prove the bound of V_k, by using (8), (35), and (19), then the time derivative of V_0 is derived as below

$$\begin{array}{c} \dot{V}_0 = -\gamma\alpha_2 s_0^2 - \frac{\beta_2}{\beta_1} I_0^2 + \frac{1}{q_c} \widetilde{\sigma}_0^2 \\ + \gamma s_0 \widetilde{\sigma}_0 f_{1,0} + \gamma s_0 \widetilde{f}_{2,0} + \frac{1}{\xi} \widetilde{f}_{2,0}^2 \end{array}. \tag{54}$$

Based on (31) and (5), $\dot{V}_0$ can be further simplified as

$$\dot{V}_0 = -\gamma\alpha_2 s_0^2 - \frac{\beta_2}{\beta_1} \beta_2 I_0^2 + \frac{1}{q_c} \widetilde{\sigma}_0^2 + \frac{1}{\xi} \widetilde{f}_{2,0}^2 \ . \tag{55}$$

Therefore, $\dot{V}_0$ is bounded in the interval of $[t_0,\, t]$. Due to $V_0(t) = V_0(t_0) + \int\limits_{t_0}^{t} \dot{V}_0(s) ds$, V_0 is also bounded in $[t_0,\, t]$. It follows from (52) that V_k is bounded. Accordingly, I_k, $\widetilde{Q}_{j,k}$, $\widetilde{\sigma}_k$, $\widetilde{f}_{2,k}$, and s_k are also bounded. Furthermore, based on the (52), it can be written that

$$\int\limits_{t_0}^{t} s_k^2 d\tau \leq \frac{1}{\alpha_3} (V_{k-1} - V_k). \tag{56}$$

Because V_k is monotonically decreasing but it is lower bounded by zero, then the following conclusion is obtained

$$\lim_{k\to\infty}\int_{t_0}^{t} s_k^2 d\tau = 0. \tag{57}$$

Based on (35), by considering (25) and the bounds of I_k, $\widetilde{\boldsymbol{Q}}_{j,k}$, $\widetilde{\sigma}_k$, $\widetilde{f}_{2,k}$, and s_k, $\dot{s}_k$ is also bounded. Accordingly, by Barbalat-like lemma presented in Xu and Yan (2004); Sun (2009), $lim_{k\to\infty} s_k = 0$ uniformly on $[t_0, t]$. Given that the sliding surface dynamics is Hurwitz, therefore, after s_k converges to zero asymptotically, the output error converges to zero exponentially.

APPENDIX C. VIRTUAL CONSTRAINT DESIGN

This section elaborates on desired trajectory generation using virtual constraints.

$h(\theta)$ in (22) is designed using the Bezier polynomials as (Westervelt et al. (2007))

$$h(\theta) = \begin{bmatrix} b_1(w(\theta)) \\ b_2(w(\theta)) \\ \vdots \\ b_N(w(\theta)) \end{bmatrix} \tag{58}$$

where

$$b(w) = \sum_{z=0}^{Z} z!(Z-z)!w^z(1-w)^{Z-z}. \tag{59}$$

In (59) Z is an integer, showing the number of Bezier polynomial terms, ϱ_k is the parameter that is going to be optimized, and w is calculated according to the following equation

$$w(\theta) = \frac{\Theta(\theta) - \Theta^+}{\Theta^- - \Theta^+} \tag{60}$$

where Θ^+ and Θ^- are maximum value and minimum value of the $\Theta(\theta)$. $\Theta(\theta)$ is defined as

$$\Theta(\theta) = \zeta_1\theta_1 + \zeta_2\theta_2 + ... + \zeta_n\theta_N \tag{61}$$

where $\zeta_i \in \mathbb{R}$ should be chosen such that $\Theta(\theta)$ is monotonically increasing. $\theta_1, \theta_2, ...\theta_N$ represents for joints $1, 2, ...N$ angular positions. Optimal Bezier polynomial coefficients were found by an evolutionary optimization algorithm (Molazadeh et al. (2019)). The cost function is defined to perform the optimization process that finds virtual constraints resulting in minimum required input torque. The optimization algorithm is depicted in Fig. 1. In the first step, the optimization is performed offline based on the system model. The computed virtual constraint is embedded in the controller as the desired profile.

In this study, a sit-to-stand task was chosen for investigating the performance of the controller. For this task, the knee joints' trajectories change monotonically, either decreasing or increasing. Therefore, both the left or right knee joint angle can be selected as a phase variable. Each leg cannot have an independent phase variable because it causes misalignment and miscoordination between the left and right legs. Therefore we only selected the right knee joint angle as the phase variable to coordinate both legs' joints in this study. Additionally, because all joints are actuated, one of the joints (here the right knee) follows a predesigned

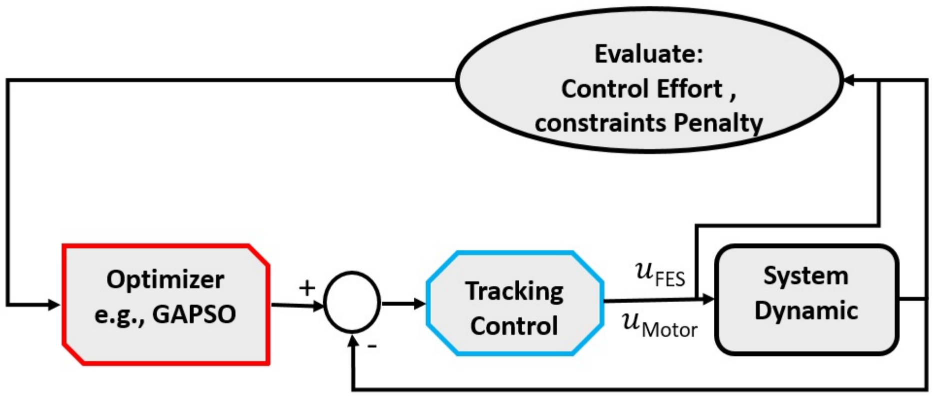

Figure 1. The optimization algorithm to determine the optimal Bezier polynomial coefficients

time-dependent trajectory. All other joints use the state-dependent virtual constraint that is a function of the actual right knee angular position (phase variable). It should also be noted that the right knee desired velocity is also a function of the right knee phase variable.

APPENDIX D. MPC ALLOCATION ALGORITHM

The steps of the model predictive allocation algorithm can be found in Table 1.

Table 1. Steps of model predictive allocation strategy

**1**	**Initialization:** $r = 0$
(1a)	The convergence tolerance is set to ε_j.
(1b)	$\theta(t_r)$, $\dot{\theta}(t_r)$ are measured.
(1c)	Feedback controller and virtual constraint are used to get $\bar{h}_k$, and total torque demand, where $\tau_k \in [t_r, t_r + t_p]$.
(1d)	An initial control trajectory is chosen $\bar{u}_{F,j,k}(\tau_k) \in \mathcal{U}_{[t_r, t_r+t_p]}$, where $\tau_k \in [t_r, t_r + t_p]$.
(1e)	$\bar{u}_{F,j,k}(\tau_k)$ and $\bar{h}_k(\tau_k)$ are used for obtaining $\bar{\tau}_{F,j,k}(\tau_k)$ and $J^{(r)}_{mpc}(t_r)$, where $\tau_k \in [t_r, t_r + t_p]$.
**2**	**Optimal Solution Searching:**
(2a)	For solving the costates, integration backward in time is done for $l^{(r)}_j(\tau)$ $H = J^{(r)}_{mpc} + \sum_{j=1}^{N_f} l^{(r)T}_j \Phi_{\mu,j}$, so the optimal solution is given $\dot{l}^{(r)}(\tau) = -\frac{\partial H(z, l^{(r)}, \bar{u}_F)}{\partial z}$, where $z = [\bar{x},\ \bar{\mu}]$ and $\Phi_{\mu,j} : \dot{\bar{\mu}}_{j,k} = \frac{(\mu_{min,j} - \bar{\mu}_{j,k})\bar{u}_{F,j,k}}{\tau_{f,j}} + \frac{(1-\bar{\mu}_{j,k})(1-\bar{u}_{F,j,k})}{\tau_{r,j}}$.
(2b)	The search direction, $a^{(r)}(\tau)$, is computed from the Hamiltonian $a^{(r)}_j(\tau) = -\frac{\partial H(z, l^{(r)}, \bar{u}_{F,j,k})}{\partial \bar{u}_{F,j,k}}$.
(2c)	The optimal step size, $\sigma^{(r)}$, is computed with an adaptive setting.
(2d)	The control trajectory is updated. $\bar{u}^{(r+1)}_{F,j,k}(\tau) = \Phi_{j,k}(\bar{u}^{(r)}_{F,j,k} + \sigma^{(r)} a^{(r)}_j)$, where the constraints are denoted by $\Phi_{j,k}$.
(2e)	$\bar{u}^{(r+1)}_{F,j,k}$ is used to get $J^{(r+1)}_{mpc}(t_r)$.
(2f)	Quit conditions are checked (i) if $\left| J^{(r+1)}_{mpc}(t_r) - J^{(r)}_{mpc}(t_r) \right| \leq \varepsilon_j$, quit. (ii) if r has exceeded the max iteration limit, N_t, quit. (iii) otherwise $r = r + 1$ and reiterate gradient step from (1a).

REFERENCES

Chen, M., Ge, S. S., and How, B. V. E. (2010). Robust adaptive neural network control for a class of uncertain mimo nonlinear systems with input nonlinearities. *IEEE Trans. Neural Networks* 21, 796–812

Chen, W., Chen, Y.-Q., and Yeh, C.-P. (2012). Robust iterative learning control via continuous sliding-mode technique with validation on an srv02 rotary plant. *Mechatronics* 22, 588–593

Lewis, F. L., Selmic, R., and Campos, J. (2002). *Neuro-Fuzzy Control of Industrial Systems with Actuator Nonlinearities* (Philadelphia, PA, USA: Society for Industrial and Applied Mathematics)

Molazadeh, V., Zhang, Q., Bao, X., and Sharma, N. (2019). Neural-network based iterative learning control of a hybrid exoskeleton with an mpc allocation strategy. In *ASME DSCC* (ASME)

Sun, M. (2009). A barbalat-like lemma with its application to learning control. *IEEE Trans. Automat. Contr.* 54, 2222–2225

Westervelt, E. R., Grizzle, J. W., Chevallereau, C., Choi, J. H., and Morris, B. (2007). *Feedback control of dynamic bipedal robot locomotion*, vol. 28 (CRC press)

Xu, J.-X. and Yan, R. (2004). Iterative learning control design without a priori knowledge of the control direction. *Automatica* 40, 1803–1809
